# Supplementary material for: TWIST1 drives endothelial-to-mesenchymal-transition to stabilize atherosclerotic plaques
Source: Nat Commun. 2026 Feb 18;17:2905. doi: 10.1038/s41467-026-69808-z (PMC13031644; doi:10.1038/s41467-026-69808-z)

SUPPLEMENTARY INFORMATION

TWIST1 drives endothelial-to-mesenchymal-transition to stabilize atherosclerotic plaques

Blanca Tardajos Ayllon[1*], Mannekomba Diagbouga[1*], Ankita Das[1], Siyu Tian[1], Andreas Edsfeldt[2,3,4], Joanna Kalucka[5], Jovana Serbanovic-Canic[6], Emily Chambers[6], Jiangming Sun[2], Chrysostomi Gialeli[2], Mark Dunning[6], Sheila E. Francis[6], Xiuying Li[7], M Akiko Mammoto[8], Michael Simons[9], Helle F Jørgensen[10], Isabel Goncalves[2,3], Suowen Xu[11], Paul C. Evans[1§].

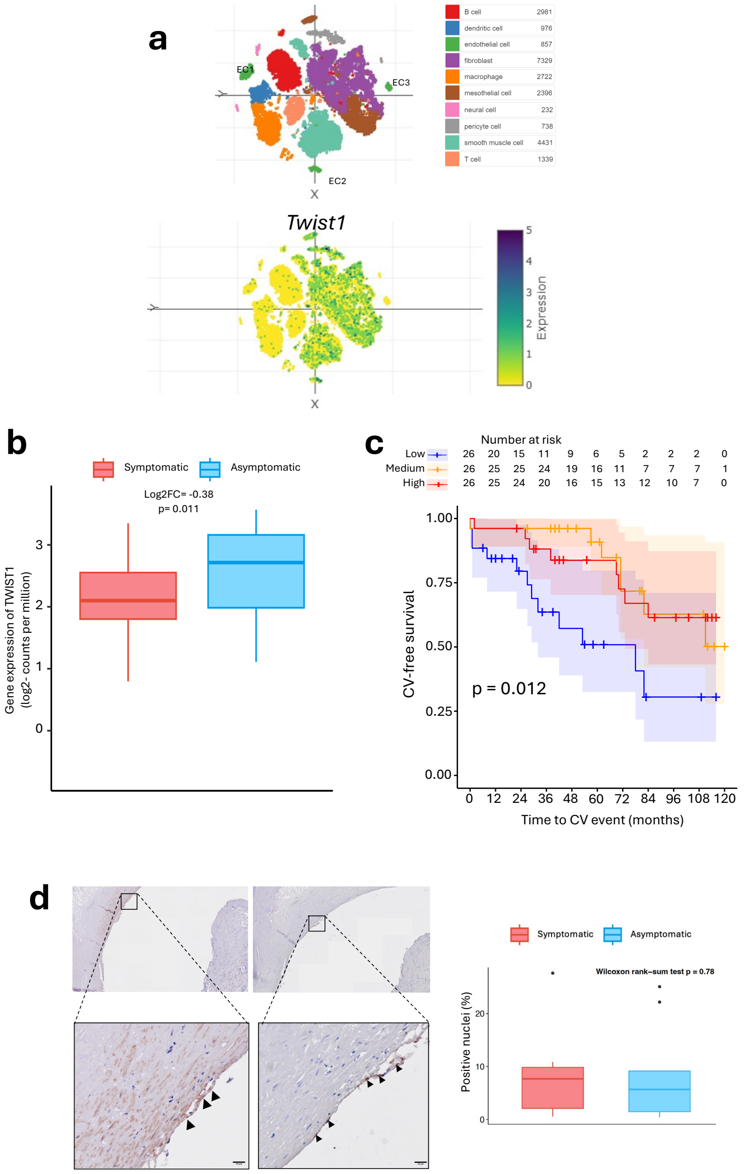

Supplementary Figure 1. Twist1 expression in murine and human plaque endothelium. (A) Published scRNAseq data were analyzed[1]. Mice were fed a Western diet for 12 weeks prior to analysis of the ascending aorta by scRNAseq (10X Genomics). Marker genes were used to define cluster identities. Three EC clusters were identified: EC1 was enriched for genes regulating lipid transport (Fabp4, Cd36, Gpihbp1), cell adhesion (Igfbp7, Cxcl12), and angiogenesis (Flt1); EC2 was enriched for canonical endothelial markers (Vcam1, Pecam1); EC3 was enriched for lymphatic markers (Lyve1, Ccl21a). (B) Twist1 expression shown as a tSNE distribution. Twist1 was detected in multiple cell types, with enrichment in fibroblasts/mesothelium, smooth muscle cells and endothelium (particularly EC2). (B, C) *TWIST1* expression levels in human carotid artery endarterectomies were analyzed using bulk RNA sequencing. (B) *TWIST1* levels were significantly lower in plaques from symptomatic patients (n=51) compared to asymptomatic ones (n=27). (B) Kaplan–Meier survival curves showing that lower plaque *TWIST1* mRNA levels (1st tertile compared to 2nd-3rd tertile of *TWIST1* mRNA levels) predict post-operative cardiovascular events. (D) TWIST1 expression levels in human carotid artery endarterectomies were quantified by immunohistotochemistry. Two representative cases are shown with staining for TWIST1 (brown) with nuclei counterstained using H&E. Lower panels show a magnified view of the boxed region. Arrows indicate TWIST1 expression in ECs. Analysis was performed using QuPath (v0.6.0).[2] Plaque cells were segmented using StarDist[3] model and TWIST1-positive nuclei were identified based on DAB intensity thresholds. No differences in TWIST1 levels were detected between asymptomatic (N=9) versus symptomatic (N=10) plaques.

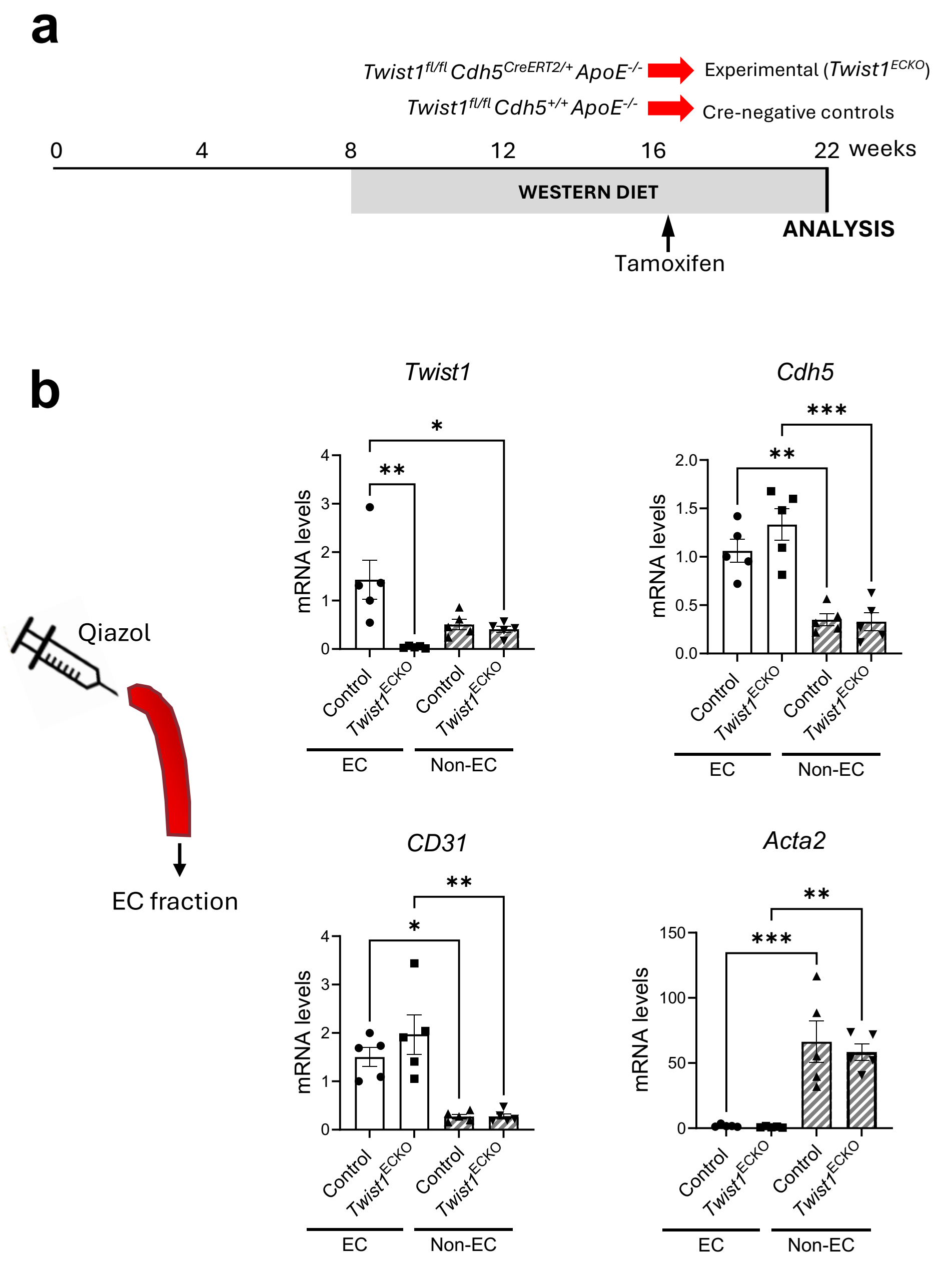

Supplementary Figure 2. Validation of *Twist1* knock-down in mouse aorta. (A) Timeline of *Twist1* deletion in a model of atherosclerotic progression. *Twist1*^{ECKO} (*Twist1*$^{fl/fl}$ *Cdh5*$^{CreERT2/+}$ *ApoE*$^{-/-}$) and control mice (*Twist1*$^{fl/fl}$ *Cdh5*$^{+/+}$ *ApoE*$^{-/-}$) aged 8 weeks were fed a Western diet for 8 weeks to induce atherosclerotic lesions. At that point, tamoxifen was administered for 5 consecutive days to induce *Twist1* deletion and a Western diet was provided for an additional 6 weeks (totalling 14 weeks of Western diet). (B) Validation of *Twist1* deletion in the endothelium. RNA was extracted from EC fractions (by flushing of Qiazol) and from residual medial/adventitial tissue (Non-EC) of aortas isolated from *Twist1*^{ECKO} and control mice. Expression levels of *Twist1, Cdh5, CD31* and *Acta2* were quantified by qRT-PCR (n=5). Mean values are shown +/- standard errors. Differences between means were analysed using a 2-way ANOVA.

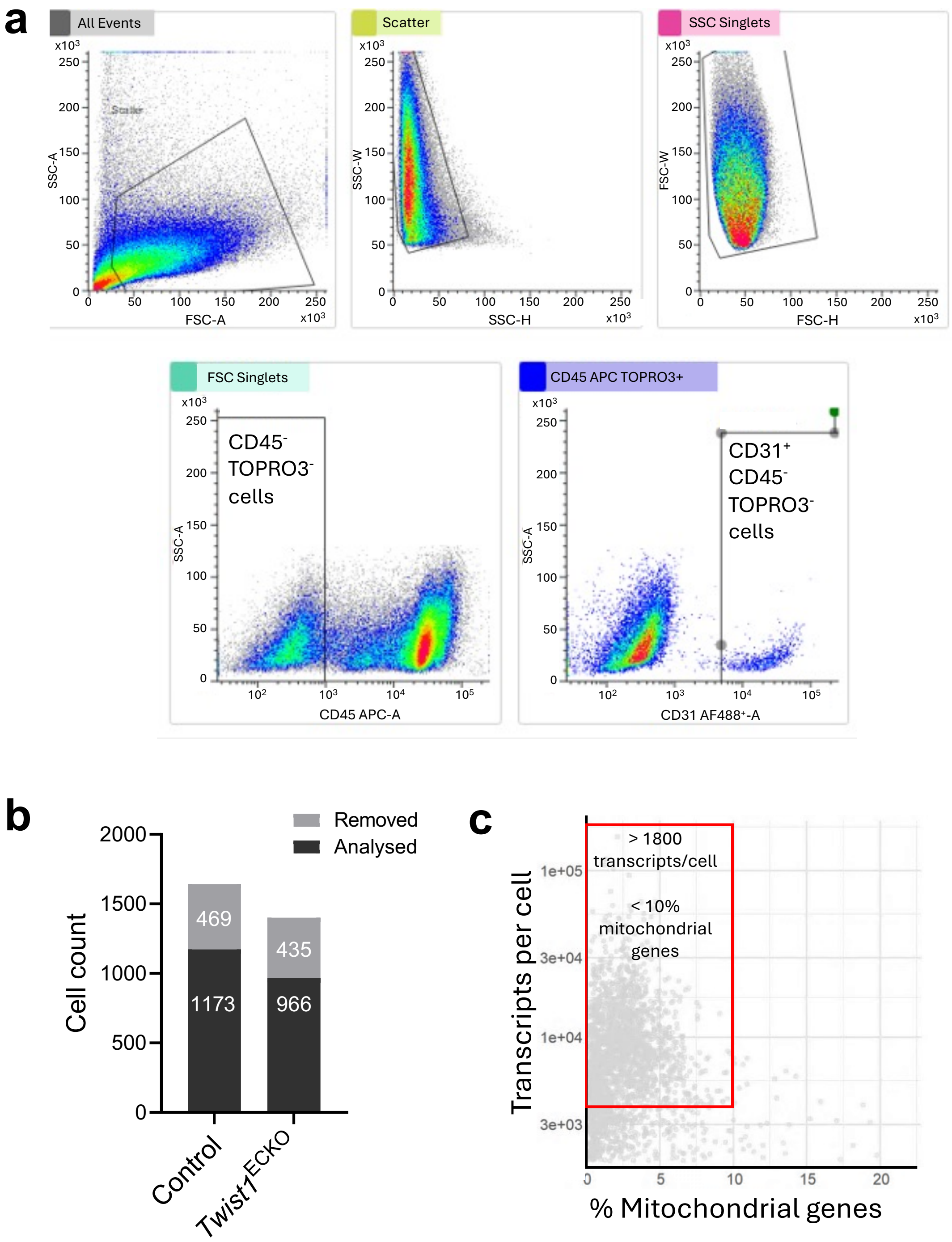

Supplementary Figure 3. scRNAseq analysis sorting strategy and quality control. Aortas from *Twist1*^{ECKO} and control mice after 14 weeks of Western diet were analysed by FACS of CD31^{+} CD45^{-} cells coupled to scRNA-seq. (A) Representative flow cytometry workflow and sorting strategy to isolate of CD31^{+}/CD45^{-}/TOPRO3^{-} cells. Single cells were identified through side scatter and forward scatter and the CD45-/TOPRO3- population was subsequently selected. From the CD45^{-}/TOPRO3^{-} population, CD31^{+} cells were sorted into a 384-well plate for scRNAseq. (B) Bar graph showing analysed cells by genotype after quality control (QC) analysis. Cells that were removed during QC analysis are represented in light grey, whereas remaining cells after QC filtering are represented in black. (C) Scatter plot showing transcripts per cell vs % Mitochondrial genes. Cells inside the red box, with <10% mitochondrial content and >1800 transcripts/cell, were selected and used for subsequent scRNAseq.

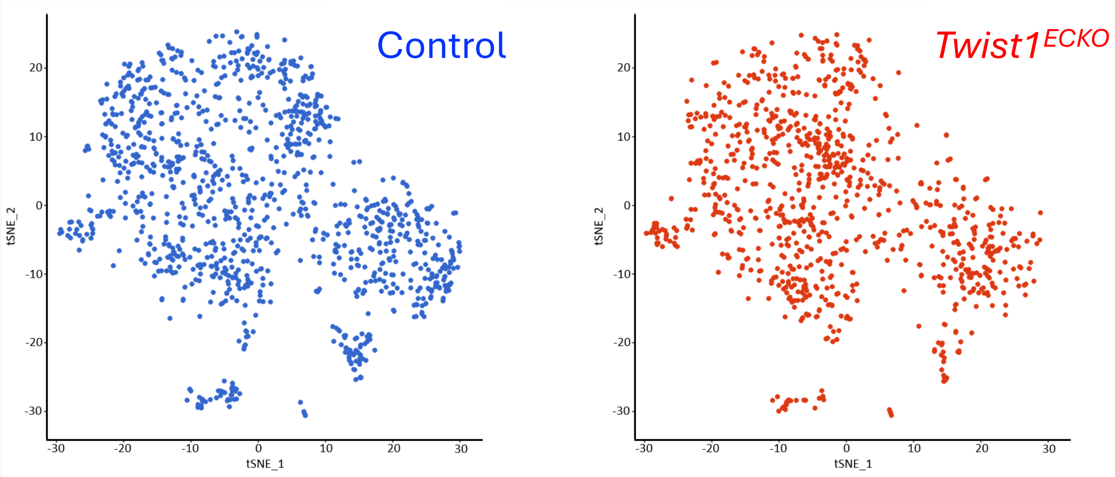

Supplementary Figure 4. EC from *Twist1^{ECKO}* and control mice exhibit different clustering patterns by scRNAseq. Aortas from *Twist1^{ECKO}* (N=4) and control (N=5) mice after 14 weeks of Western diet were analysed by FACS of CD31^{+} CD45^{-} cells coupled to scRNA-seq. t-SNE maps showing the distribution of aortic CD31^{+} CD45^{-} cells from control (left) and *Twist1^{ECKO}* (right) mice with data pooled from multiple mice.

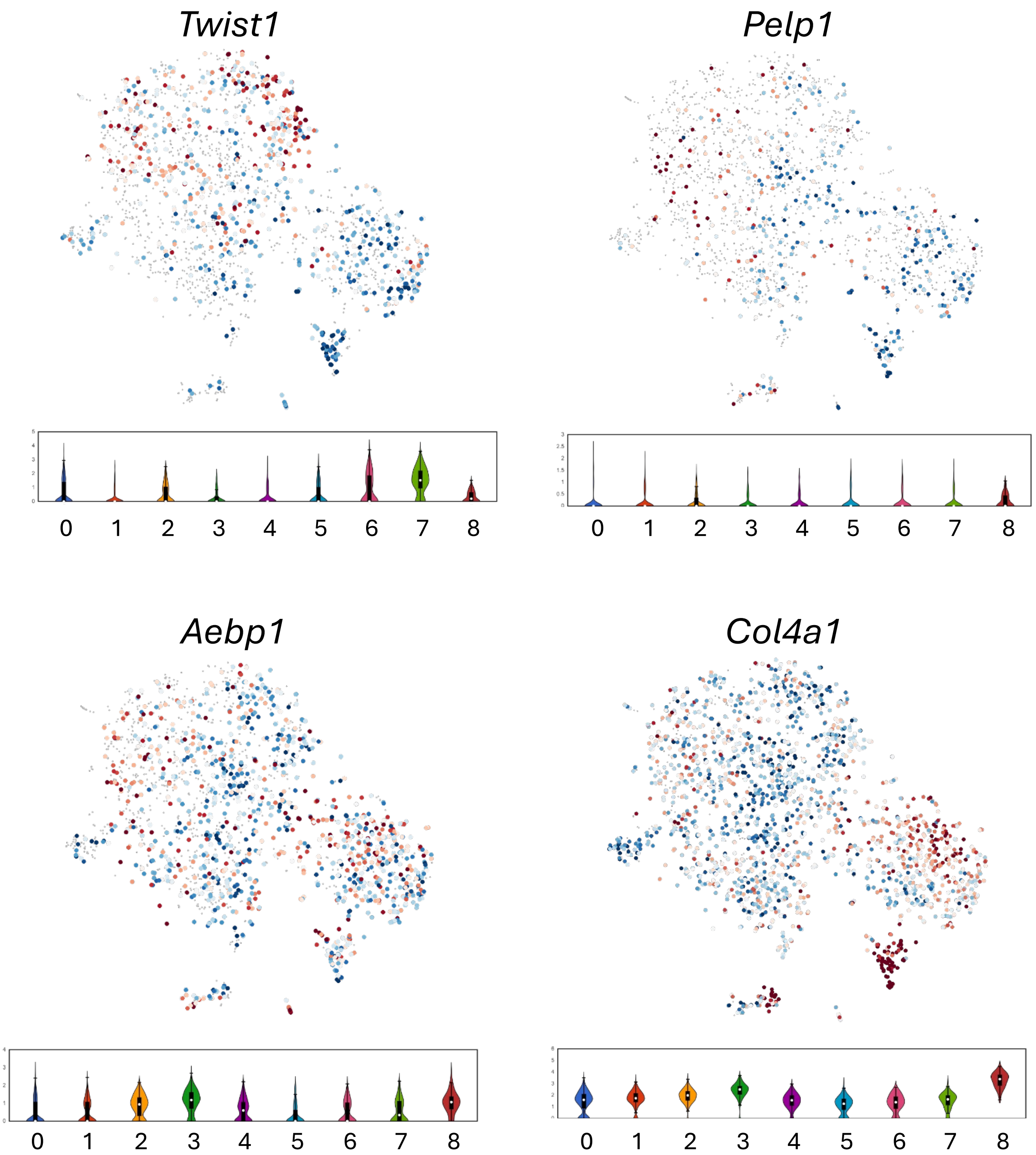

Supplementary Figure 5. Distribution of *Twist1, Pelp1, Aebp1* and *Col4a1* across scRNAseq clusters. Aortas from *Twist1*^{ECKO} (N=4) and control (N=5) mice after 14 weeks of Western diet were analysed by FACS of CD31^{+} CD45^{-} cells coupled to scRNA-seq. Expression of *Twist1, Pelp1, Aebp1* and *Col4a1* in individual cells is presented as t-SNE maps and as violin plots as an average (AUCell score).

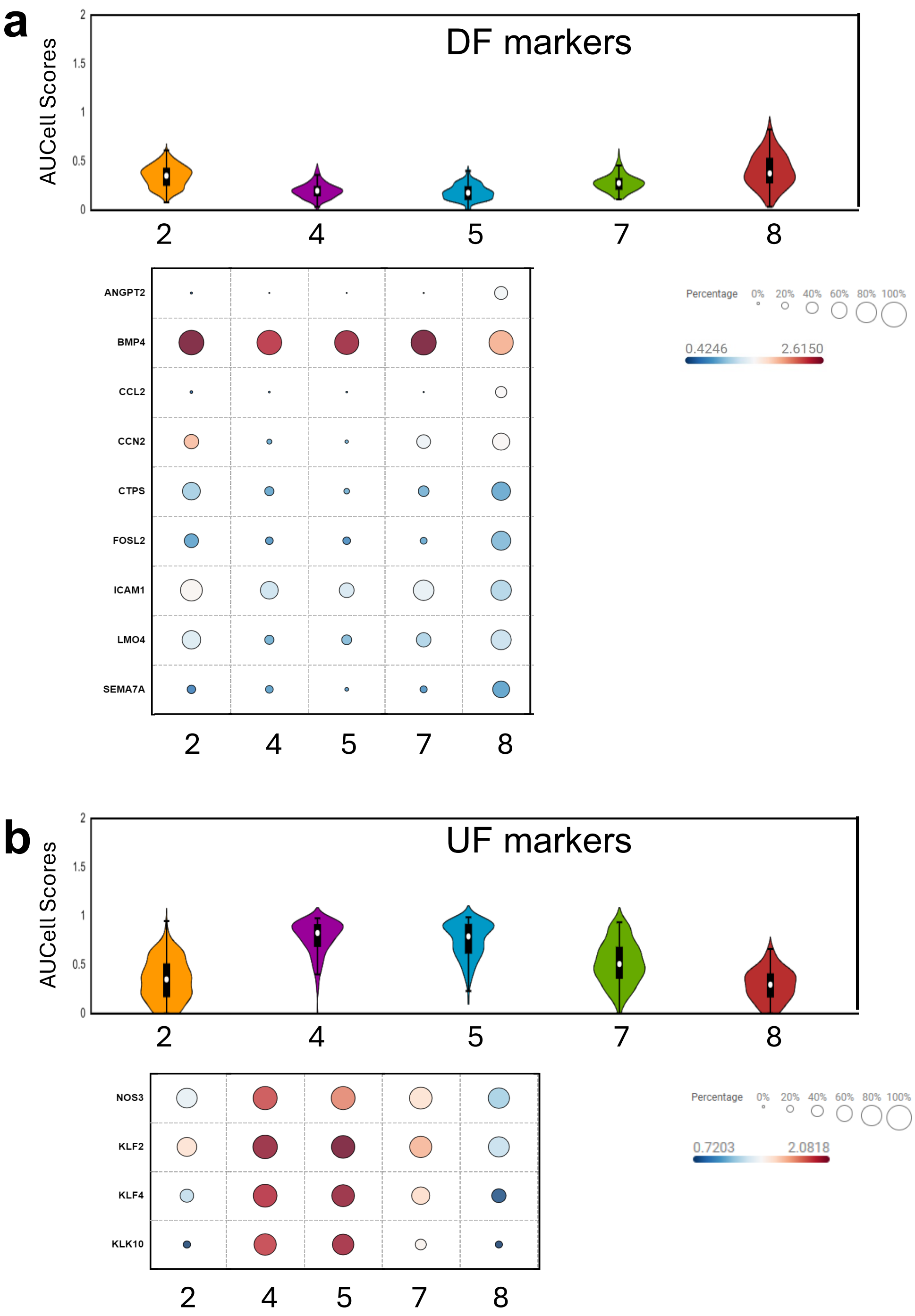

Supplementary Figure 6. Expression of uniform flow and disturbed flow markers in selected scRNAseq clusters. After 14 weeks of Western diet, CD31^{+} CD45^{-} cells from aortas from *Twist1*^{ECKO} and control mice were processed for scRNA-seq. (A) DF markers were measured in each cell. At the top, DF markers are presented as a violin plot as an average in selected clusters. At the bottom, DF markers are presented individually as a bubble plot in selected clusters. (B) Uniform flow (UF) markers were measured in each cell. At the top, UF markers are presented as a violin plot as an average in selected clusters. At the bottom, UF markers are presented individually as a bubble plot in selected clusters. Clusters 2, 7, 8 and 9 (largely composed of ECs derived from control mice) and 4 and 5 (mainly composed of ECs derived from *Twist1*^{ECKO} mice were compared.

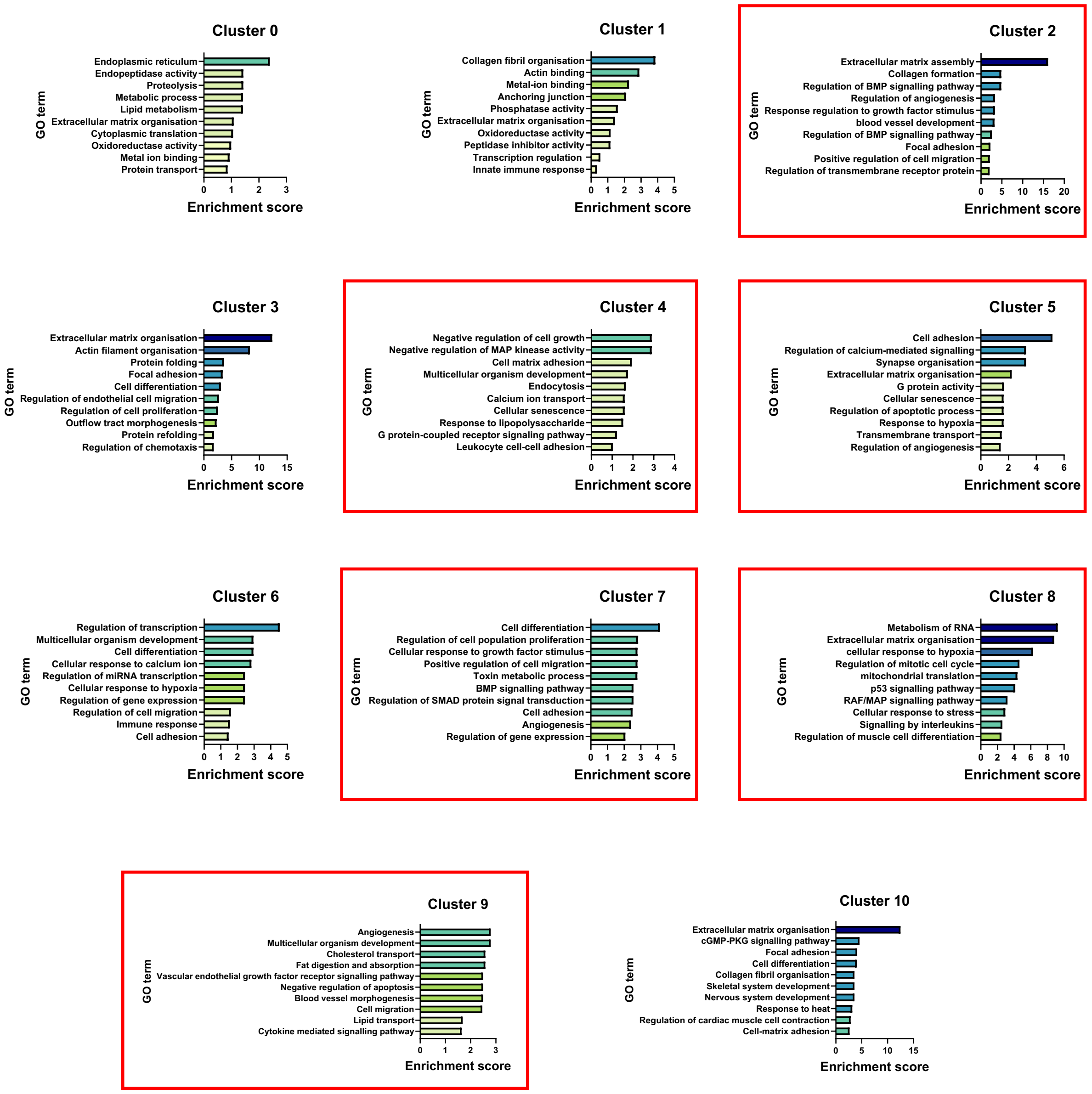

Supplementary Figure 7. Functional annotation of scRNAseq. scRNA-seq analysis of CD31$^+$ CD45$^-$ cells from *Twist1^{ECKO}* and control mice revealed 11 distinct clusters. The most highly enriched GO pathways for each cluster, as well as their enrichment scores, are shown. Clusters 2, 7, 8 and 9 (largely composed of ECs derived from control mice) and 4 and 5 (mainly composed of ECs derived from *Twist1^{ECKO}* mice) are highlighted in red.

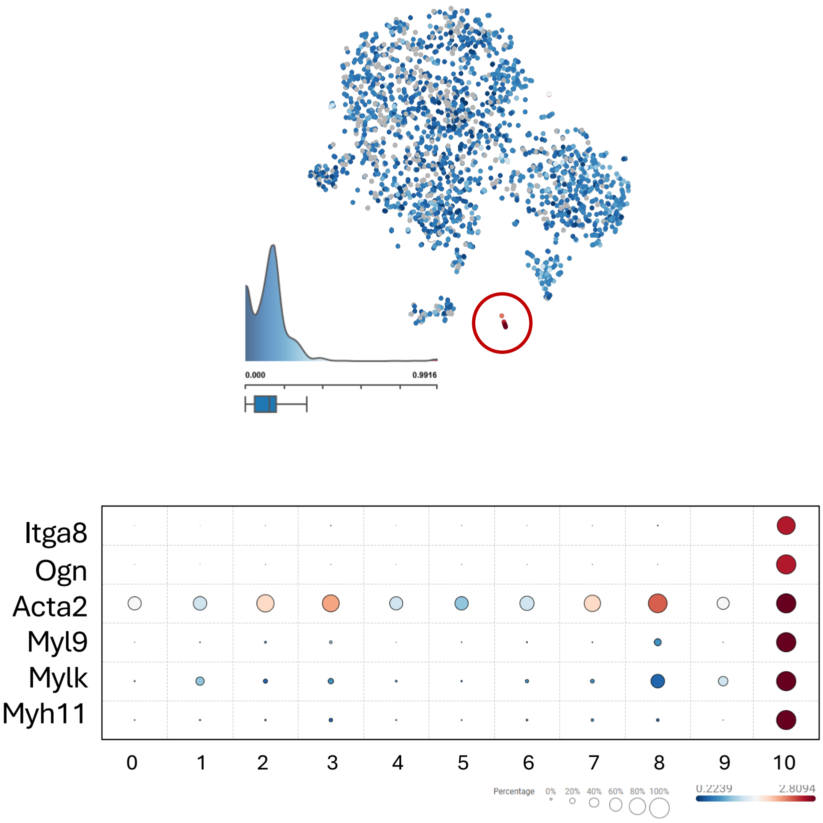

Supplementary Figure 8. Expression of enriched markers in scRNAseq cluster 10. scRNA-seq analysis of CD31^{+} CD45^{-} cells from *Twist1^{ECKO}* and control mice revealed 11 distinct clusters. t-SNE map and bubble map indicating the expression of the most highly enriched markers in cluster 10, which is circled in red on the t-SNE map.

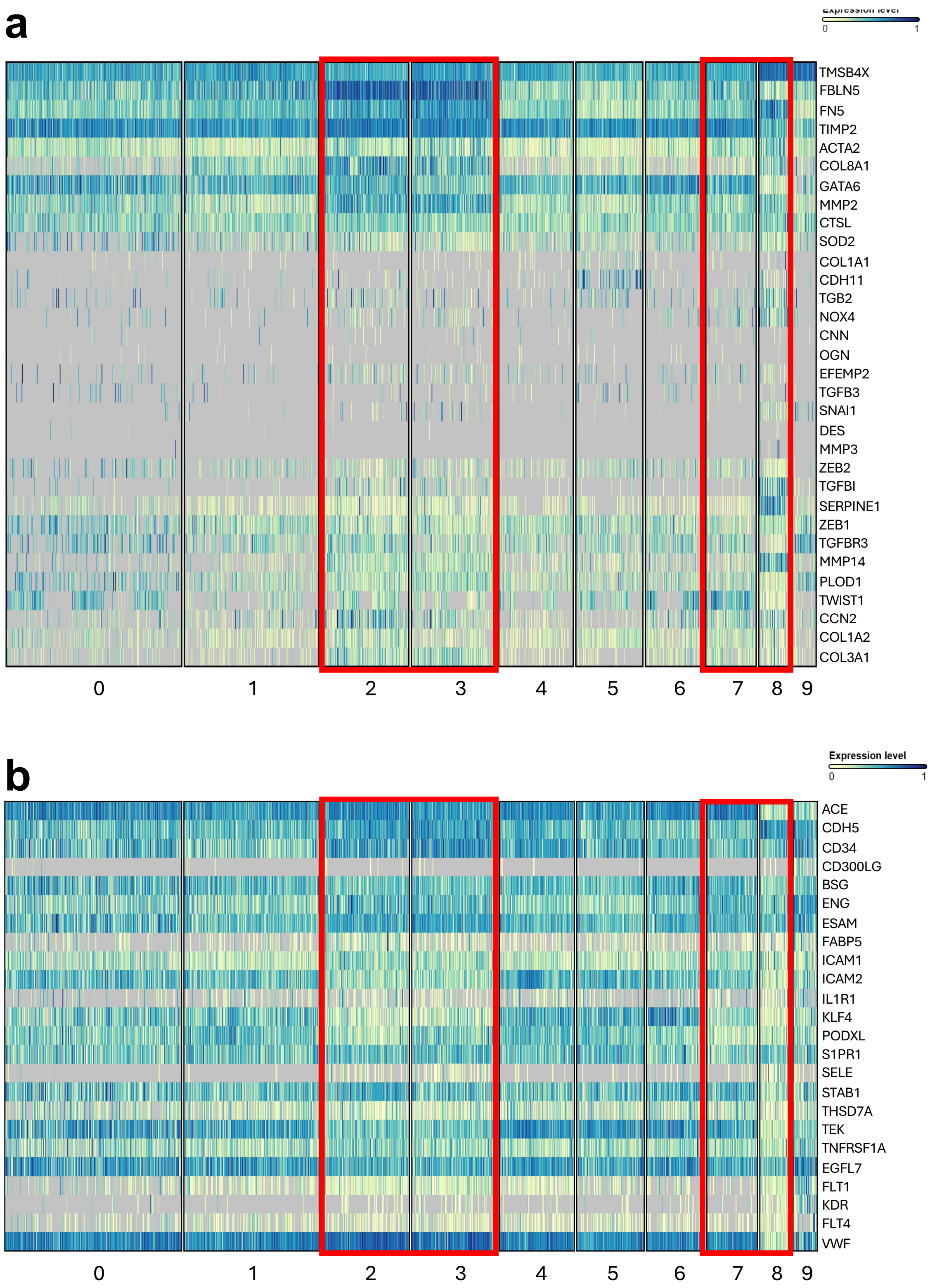

Supplementary Figure 9. **Expression of markers of EndMT-like cells and ECs markers in scRNAseq clusters.** Aortas from *Twist1*^{ECKO} and control mice after 14 weeks of Western diet were analysed by FACS of CD31^{+} CD45^{-} cells coupled to scRNA-seq. (A) Heatmap showing expression of markers of EndMT in clusters 0-9 (EC clusters). (B) Markers of ECs in clusters 0-9. Clusters 2, 3, 7 and 8, which show enriched expression of EndMT markers, are highlighted in red.

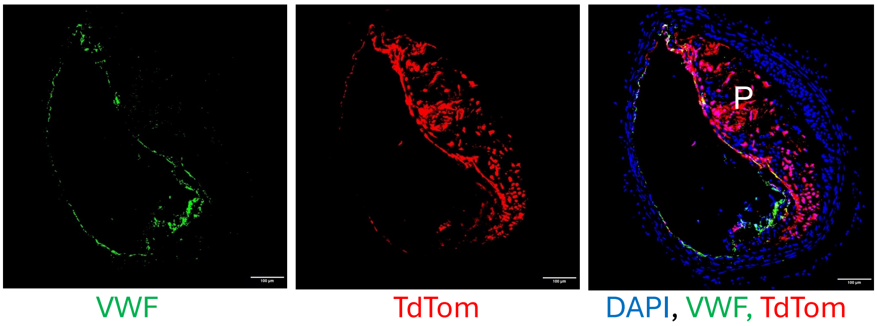

Supplementary Figure 10. TdTomato correlates with vWF expression in EC tracking transgenic mice. *Cdh5*$^{CreERT2/+}$ *ApoE*$^{-/-}$ *Rosa26*$^{TdTomato/TdTomato}$ mice aged 8 weeks were fed a Western diet for 8 weeks to induce atherosclerotic lesions. Tamoxifen was then administered for 5 consecutive days to induce *Twist1* deletion and TdTomato expression in ECs and a Western diet was provided for an additional 6 weeks (totalling 14 weeks of Western diet). Frozen sections of brachiocephalic arteries were stained using antibodies against VWF (green). Rosa26TdTomato^{+} cells are shown in red, and nuclei are counterstained with DAPI (blue). Rosa26TdTomato^{+} cells correlate spatially with vWF+ EC at the lumen, and accumulate within the plaque. Representative images are shown (Scale bar=100 μm).

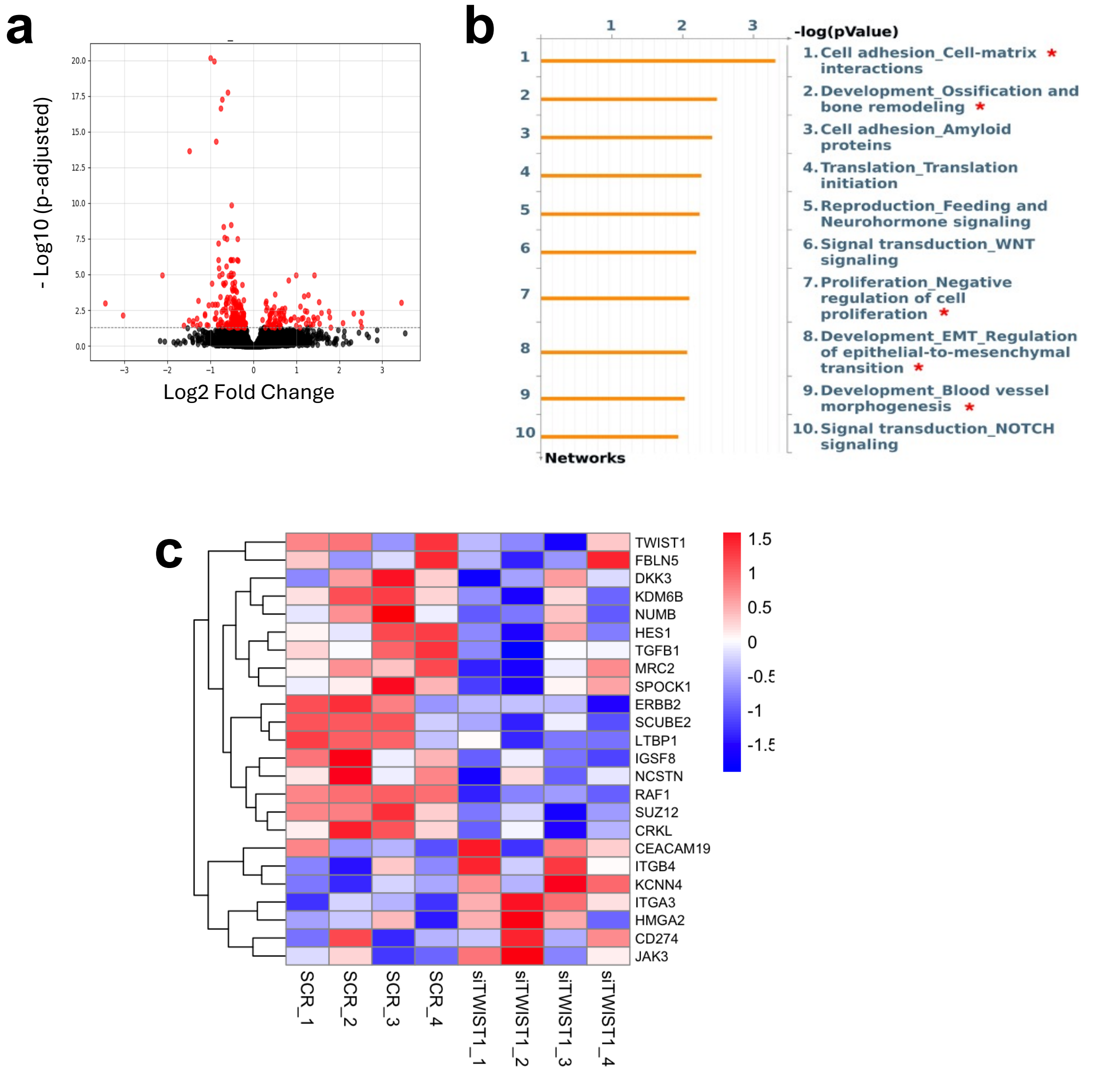

Supplementary Figure 11. TWIST1 siRNA reduces EndMT marker expression in DF conditions. HAECs were transfected with *TWIST1* siRNA (*siTWIST1(v1)*) or scrambled (SCR) siRNA before exposure to DF for 72h (Ibidi system) and analysis by RNAseq. (A) Volcano plot of RNA-seq data (n= 4). Differentially expressed genes (Padj<0.05) are highlighted in red. (B) Process Network Analysis (MetaCore) identified biological processes enriched by *TWIST1 siRNA* in RNA-seq. Pathways related to EndMT or cell proliferation are indicated by red asterisks. (C) Heatmap showing expression of markers of EndMT. Gene expression levels were reported as fragments per kilobase of transcript per million mapped reads. Data were log_2-transformed with a pseudocount (+0.1) and Z-score normalized across each gene (row-wise) to emphasize relative expression changes between donors. The heatmap was generated in R using the pheatmap package.

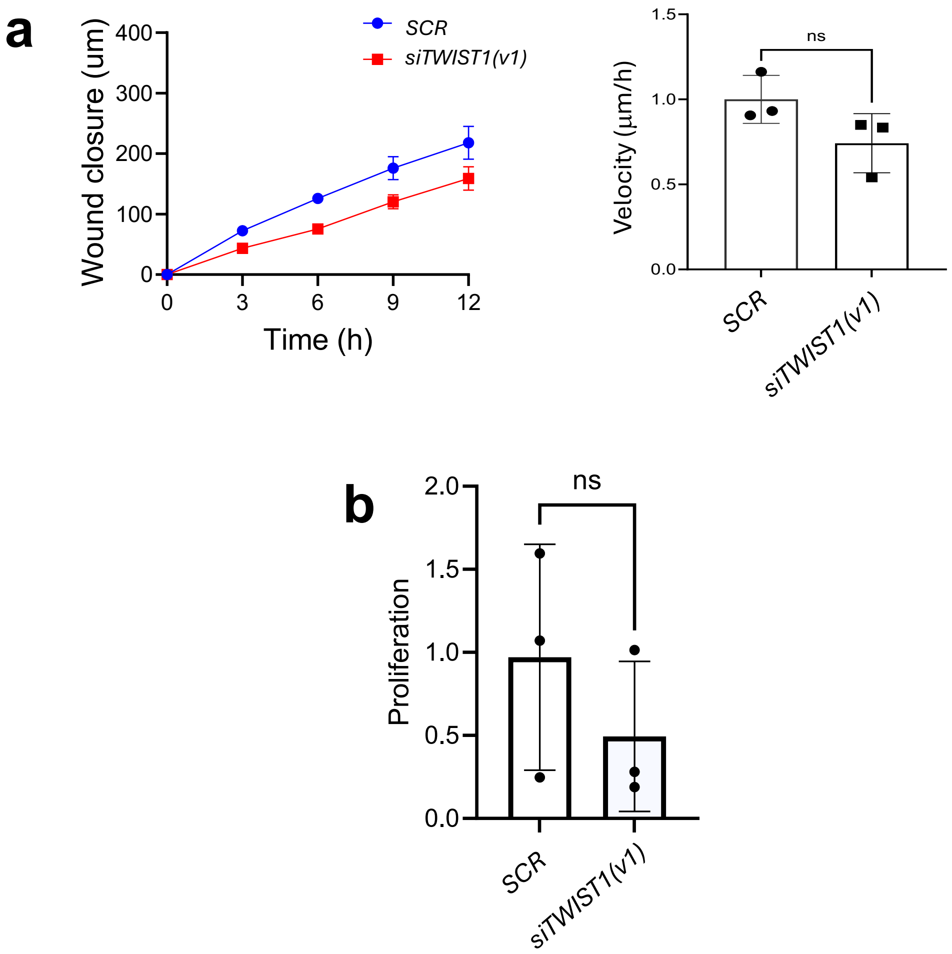

Supplementary Figure 12. *TWIST1* silencing has modest effects on migration and proliferation in arterial EC exposed to UF. (A) Cell migration was assessed using a scratch wound assay in *SCR* vs *siTWIST1(v1)* HAEC monolayers after exposure to UF (orbital shaker). (A) The distance migrated from the initial wound (T0) was measured at multiple time points. Average migration velocity over 12 hours was quantified (distance migrated/12h) (n=3). (B) Ki67 immunofluorescence staining (red) was performed to quantify proliferation in *SCR* vs *siTWIST1(v1)*-treated HAEC under UF (Ibidi system). Ki67-positive cells were quantified as a proportion of total nuclei and values are shown as relative fold change compared to *SCR*.

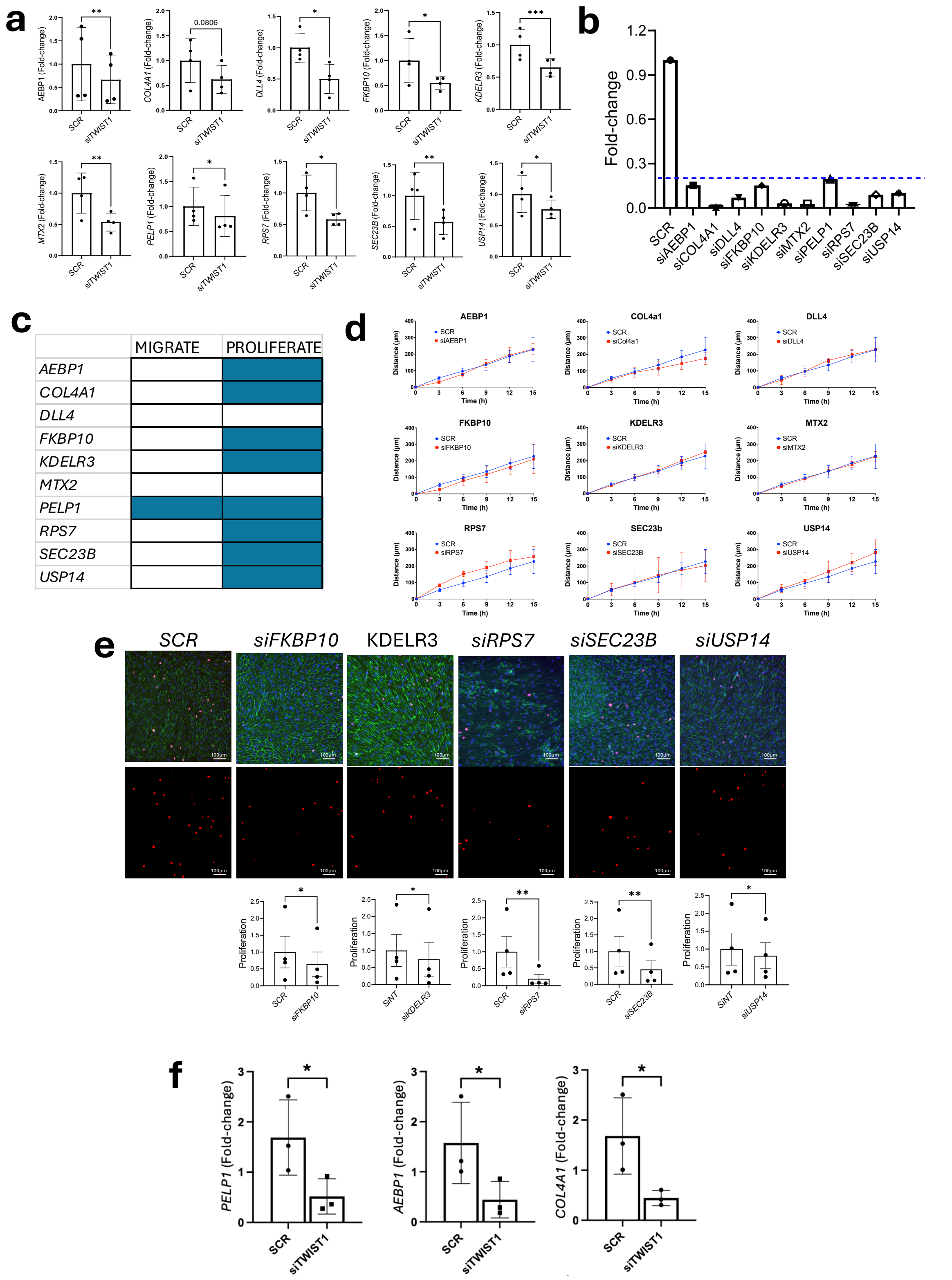

Supplementary Figure 13. TWIST1 regulated genes are involved in cell migration and proliferation. (A) qPCR validation of RNA-seq data. Gene expression levels of *AEBP1*, *COL4A1*, *DLL4*, *FKBP10*, *KDELR3*, *MTX2*, *PELP1*, *RPS7*, *USP14*, and *SEC23B* in si*TWIST1(v1)*-silenced HAECs compared to control (*SCR*) cells (n=4). (B) qPCR confirmation of siRNA-mediated silencing of these genes. (C) Summary table categorizing genes based on their involvement in proliferation and/or migration. (D) Cell migration was assessed using a scratch wound assay in HAEC monolayers transfected with siRNA targeting *AEBP1*, *COL4A1*,

DLL4, *FKBP10*, *KDELR3*, *MTX2*, *RPS7*, *USP14*, and *SEC23B,* or a non-targeting control (SCR) after exposure to DF for 72h (orbital shaker). Quantification of migration distance from the initial wound (T0) at multiple time points is shown. (E) Ki67 immunofluorescence staining (red) was performed to assess proliferation after siRNA-mediated silencing of *FKBP10-*, RPS7 and *SEC23* in HAECs exposed to DF for 72h (orbital shaker). Merged images show DAPI (blue) and CDH5 (green) (scale bar=100 μm). Ki67-positive cells were quantified as a proportion of total nuclei and values are shown as relative fold change compared to *SCR* (n=4). (F) HAEC were treated with *siTWIST1(v2)* and exposed to DF for 72 h prior to qRT-PCR analysis for *PELP1, AEBP1* and *COL4A1*. Mean values are shown +/- standard errors. Differences between means were analysed using a ratio paired t-test.

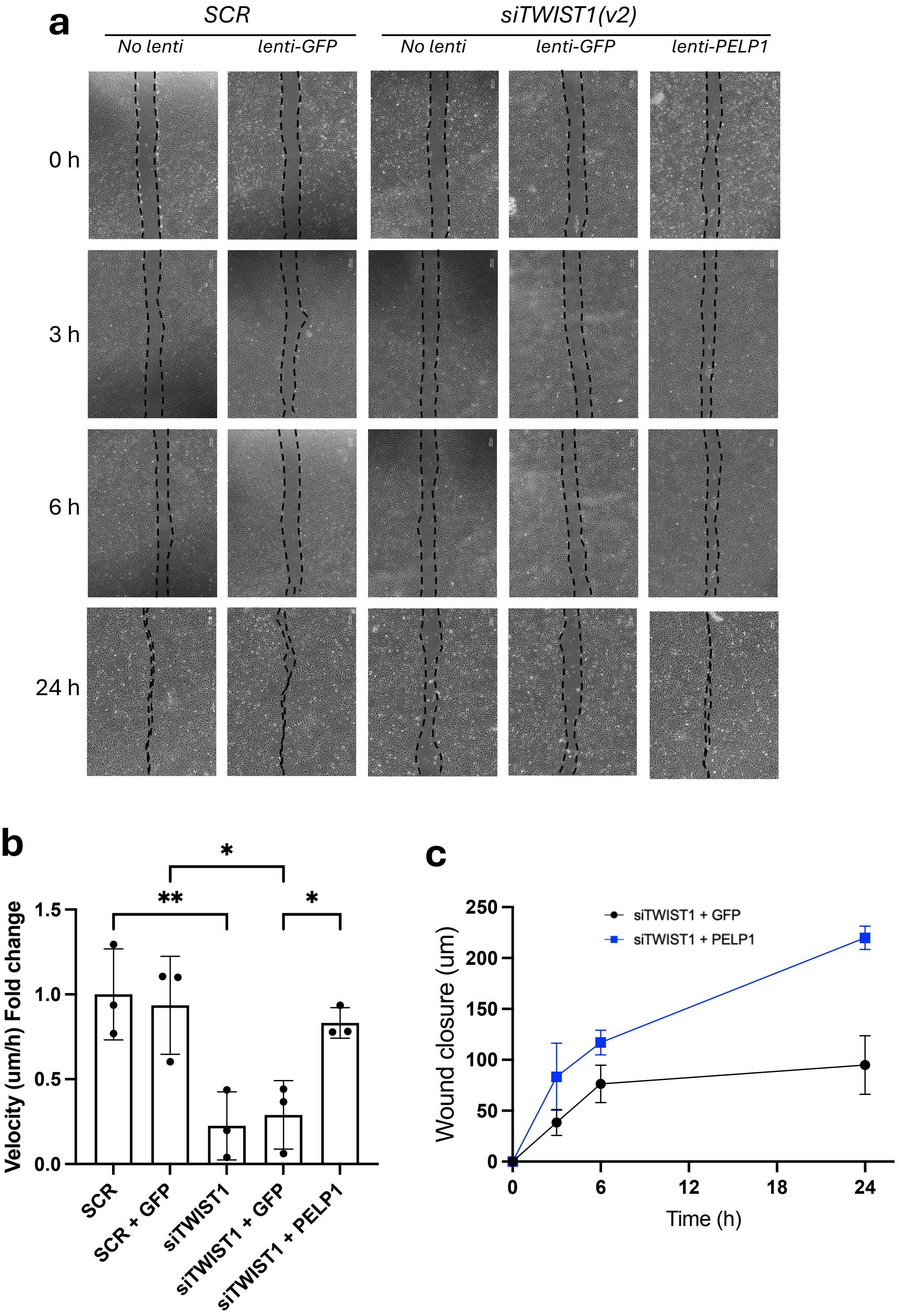

Supplementary Figure 14. *PELP1* expression rescues migration in *TWIST1*-silenced EC. HAECs were transfected with *TWIST1* siRNA (*siTWIST1(v2)*) or scrambled (*SCR*) siRNA in the presence of lentivirus containing *PELP1* cDNA (*lenti-PELP1*), *GFP* cDNA (*lenti-GFP*) or without lentivirus as a control (*No lenti*). Cell migration was assessed using a scratch wound assay after exposure to DF (orbital shaker). (A) Brightfield images show wound closure at different time points (scale bar= 200 µm). (B) Average migration velocity over 24 h was quantified (distance migrated/ 24 h) (n=3). Mean values are shown +/- standard errors. Differences between means were analysed by two-way ANOVA. (C) The distance migrated from the initial wound (0 h) was measured at multiple time points.

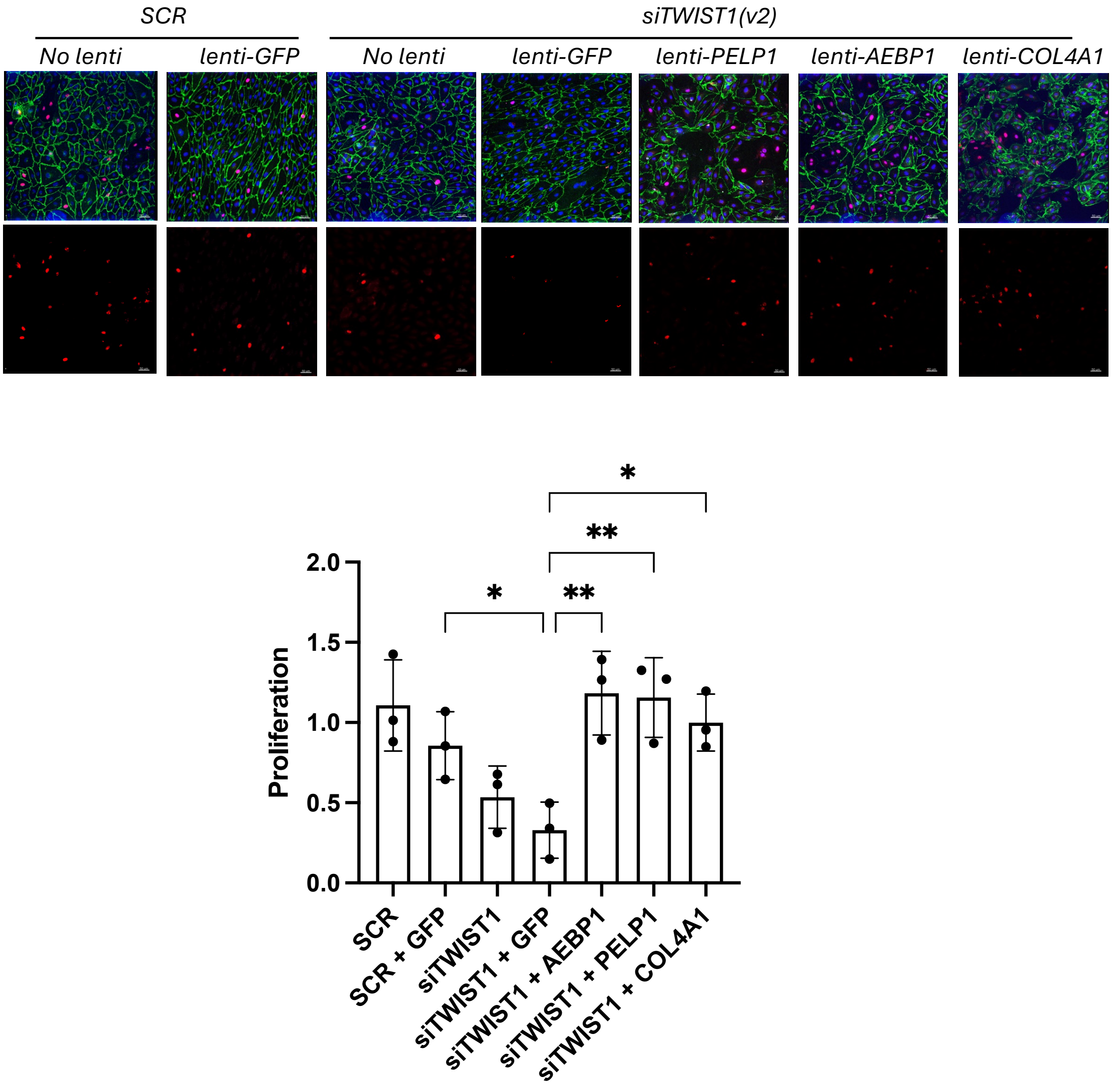

Supplementary Figure 15. Expression of *PELP1*, *AEBP1* or *COL4A1* rescues migration in *TWIST1*-silenced EC. HAECs were transfected with *TWIST1* siRNA (*siTWIST1(v2)*) or scrambled (*SCR*) siRNA in the presence of lentivirus containing *PELP1, AEBP1*, or *COL4A1* cDNA, or *GFP* cDNA as a control. Cultures were exposed to DF for 72 h. Ki67 immunofluorescence staining (red) was performed. Merged images show DAPI (blue) and CDH5 (green) (scale bar= 50 µm). Ki67-positive cells were quantified as a proportion of total nuclei and values are shown as relative fold change compared to *SCR*. Mean values are shown +/- standard errors. Differences between means were analysed by two-way ANOVA.

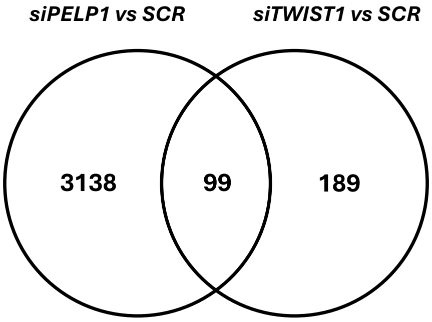

99 genes co-regulated by PELP1 and TWIST1

ADPRH	CMTM3	FRS2	LOC64413	OAS2	RNF152	VPS54
AEBP1	CNOT6	**GRB10**	5	P3H1	RPS25	WASHC4
**ANXA3**	COL4A1	GSDMC	LRRC8A	**PELP1**	**SEMA3A**	WDFY4
ATP9A	CREBRF	HADHA	MAGED4B	PHYH	SLC12A2	ZNF24
BCL7A	**CRKL**	**HES1**	MAP1A	PLA2G12A	SLC35D1	
BDH1	**CSF1**	HLA-DRB1	MAPK6	PLIN3	SNCA	
C17orf58	CYB561A3	HMGA2	MEDAG	PNRC2	**SPOCK1**	
C5orf15	DANCR	HSPA4L	MFAP3	**PODN**	SPR	
CASP4	**DPP4**	**ICAM1**	MFNG	PPP1R18	STMN3	
CASP7	ECE1	IFI27	MRC2	**PTK7**	STRADB	
CCPG1	EEF2	IFITM3	MSL2	RABAC1	SV2A	
**CEACAM1**	EIF4EBP2	**IGSF8**	MTX2	RCC1L	SYT11	
CELF2	**EMILIN1**	**ITGB4**	NACC2	RCN3	TMEM184B	
CHST1	ERC1	KDELR3	NCSTN	RENBP	TOB1	
CLCN3	FKBP10	LAMB2	NUCB1	RFX2	TOR1A	
CLCN4	FRMD4B	LINC00520	OAS1	**RHOU**	VPS4B	

Supplementary Figure 16. TWIST1 and PELP1 co-regulate genes that promote EC migration. RNA-seq analysis was performed after siRNA-mediated silencing of *TWIST1 (siTWIST1(v1))* or *PELP1* in HAECs exposed to DF for 72h (orbital shaker). Venn diagram illustrating genes co-regulated by *TWIST1 and PELP1* and those regulated exclusively by these molecules. On the right, genes co-regulated by *TWIST1* and *PELP1* are listed with migration-associated genes highlighted in red.

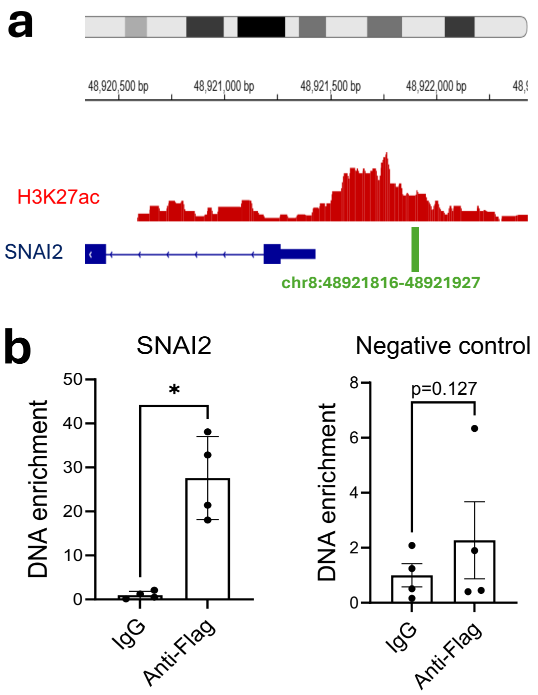

Supplementary Figure 17. ChIP-qPCR analysis of TWIST1 in HAECs under disturbed flow. HAECs were infected with lentivirus expressing TWIST1-FLAG and exposed to DF using an orbital shaker for 72h. TWIST1 binding at the *SNAI2* locus was assessed using an anti-FLAG antibody and using IgG as a control. (A) Schematic representation of the *SNAI2* gene loci, with TWIST1 binding sites highlighted in green. (B) ChIP-qPCR revealed enrichment of *SNAI2* regulatory region DNA to the IgG control (left), whereas irrelevant control sequences were not enriched (n=4). Mean values are shown +/- standard errors. Differences between means were analysed using a ratio paired t-test.

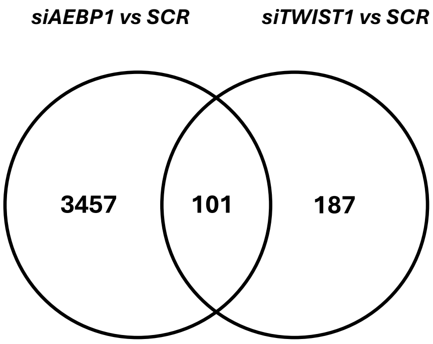

101 genes co-regulated by AEBP1 and TWIST1

ADPRH	CLCN4	EXT2	**LAMB2**	**PODN**	STMN3
AEBP1	CLN8	FAM168B	LTBP1	PREX1	SUMO3
ANXA3	CNOT6	FILIP1L	LZIC	PRX	SUPT6H
**APP**	**COL18A1**	FRMD4B	MAGED4B	PTGS1	**SUZ12**
ARSB	COL4A1	**FRS2**	MAPK6	PTK7	SV2A
ATP9A	**CRKL**	GPX7	MEDAG	RALBP1	SYT11
B3GNT9	**CSF1**	GSTM4	MFNG	**RCN3**	TMCC3
**BCL7A**	CSNK1G3	**HES1**	MRC2	RENBP	TMEM255B
BDH1	DANCR	**HMGA2**	MTX2	RPL10A	TSPAN17
C5orf15	**DLL4**	HSPA4L	**NACC2**	RPL28	UNC119B
C6orf120	DPP4	ICAM1	**NCSTN**	**SDE2**	WASHC4
CASP4	EBPL	IGF2BP2	OAS2	SEC23B	**WEE1**
CASP7	EIF4EBP2	IGSF8	**P3H3**	SEMA3A	WRNIP1
**CDK19**	**EMILIN1**	ITGB4	PAPLN	SLC12A2	ZNF532
CEACAM1	**ENSA**	KDELR3	PCSK5	SLC16A3	ZNF678
CHST1	ERC1	KDM6B	**PDCD1LG2**	SPOCK1	**ZNF703**
CHST12	ERMP1	KLF12	PLIN3	SPRY4	

Supplementary Figure 18. TWIST1 and AEBP1 co-regulate genes that promote EC proliferation. RNA-seq analysis was performed after siRNA-mediated silencing of *TWIST1 (siTWIST1(v1))* or *AEBP1* in HAECs exposed to DF for 72h (orbital shaker). Venn diagram illustrating genes co-regulated by *TWIST1 and AEBP1* and those regulated exclusively by these molecules. On the right, genes co-regulated by *TWIST1* and *AEBP1* are listed with proliferation-associated genes highlighted in blue.

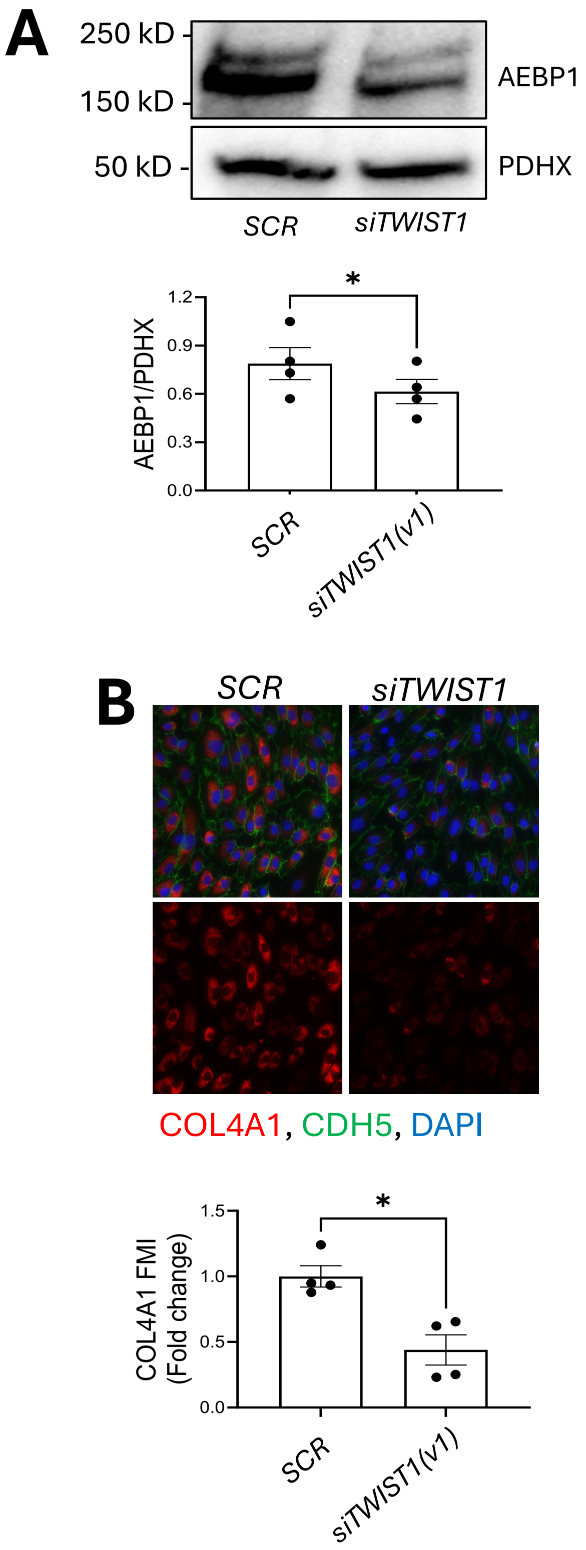

Supplementary Figure 19. TWIST1 silencing reduces AEBP1 and COL4A1 expression in HAECs under DF. (A) Western blot analysis of AEBP1 expression in *SCR* vs si*TWIST1(v1)*-silenced HAECs after 72h of DF (Ibidi system), normalized to PDHX (n = 4). (B) Immunofluorescence staining of COL4A1 (red) in HAECs after 72h of DF (Ibidi system), with merged images showing DAPI (nuclear stain, blue) and CDH5 (green) (scale bar= 100μm) (n=4). Mean values are shown +/- standard errors. Differences between means were analysed using a ratio paired t-test.

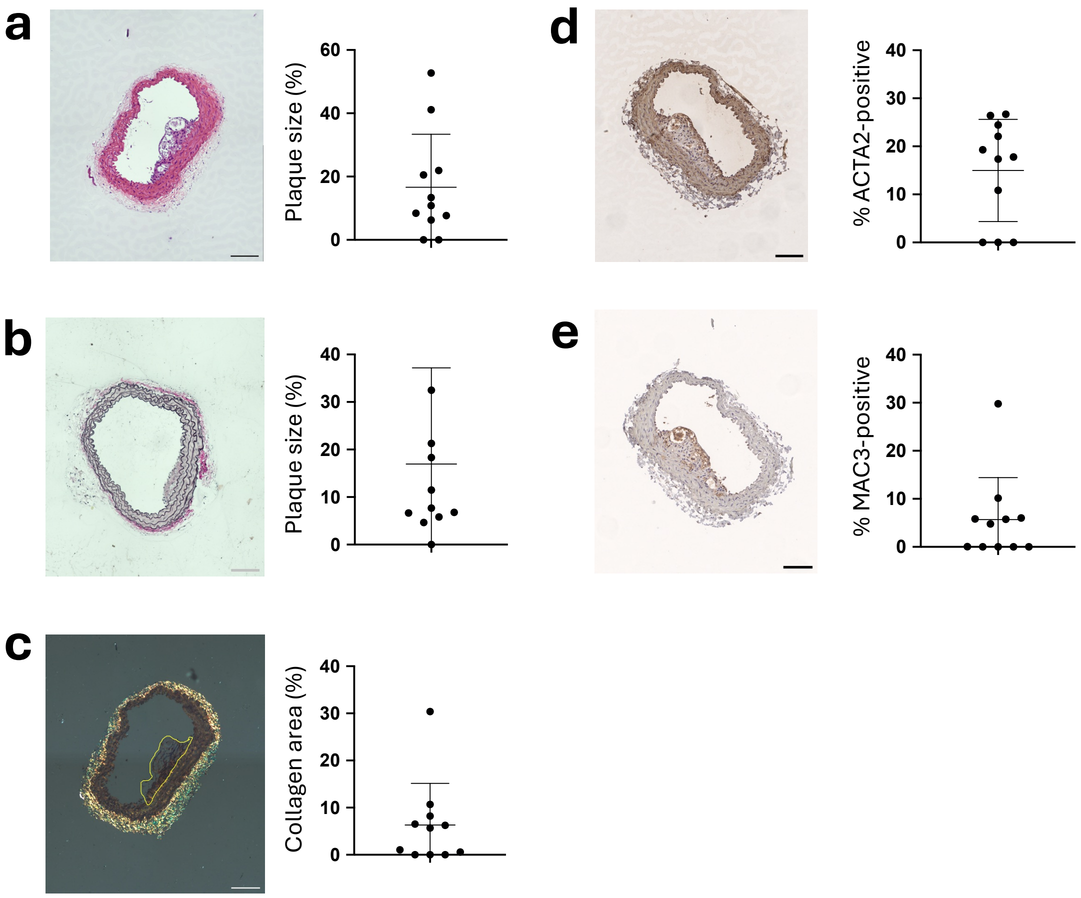

Supplementary Figure 20. Baseline measurements of brachiocephalic plaques after 8 weeks Western diet. Male *Twist1*$^{+/+}$ *ApoE*$^{-/-}$ mice aged 8 weeks (N=11) were fed a Western diet for 8 weeks. Paraffin-embedded sections of brachiocephalic arteries were stained with (A) H&E or (B) Miller's elastin stain to quantify plaque size, (C) Picrosirius Red (visualised under polarised light) to quantify collagen content, (D) antibodies against ACTA2 to quantify vSMCs content, or (E) antibodies against MAC3 to quantify macrophage content. Representative images are shown (Scale bar=100 μm). Mean values are shown +/- standard errors.

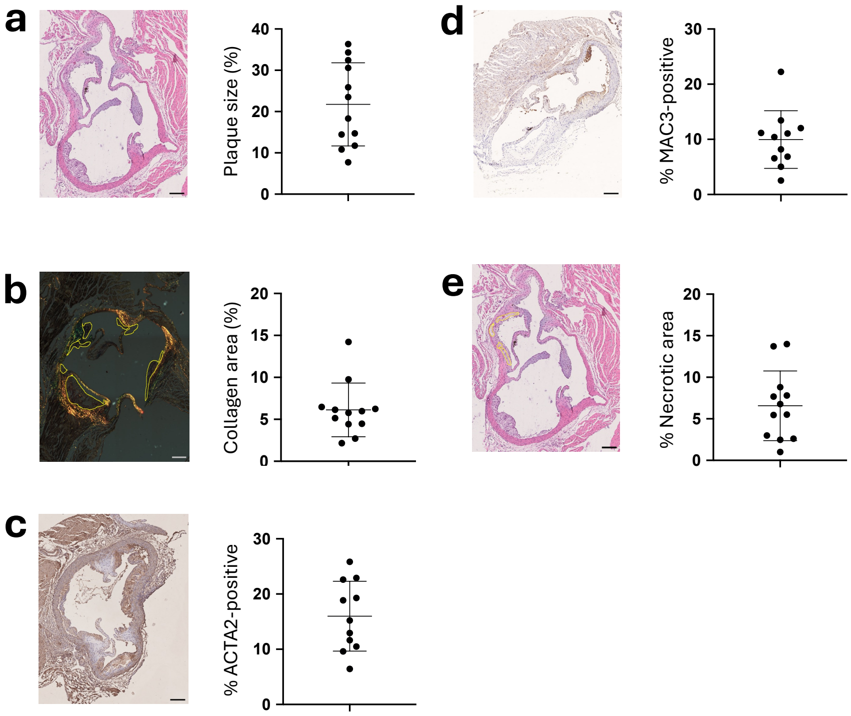

Supplementary Figure 21. Baseline measurements of aortic root plaques after 8 weeks Western diet. Male *Twist1*$^{+/+}$ *ApoE*$^{-/-}$ mice aged 8 weeks (N=11) were fed a Western diet for 8 weeks. Paraffin-embedded sections of aortic roots were stained with (A) H&E to quantify plaque size, (B) Picrosirius Red (visualised under polarised light) to quantify collagen content, (C) antibodies against ACTA2 to quantify vSMCs content or (D) antibodies against MAC3 to quantify macrophage content. (E) H&E staining was also used to identify and quantify necrotic areas. Representative images are shown (Scale bar=100 μm). Mean values are shown +/- standard errors.

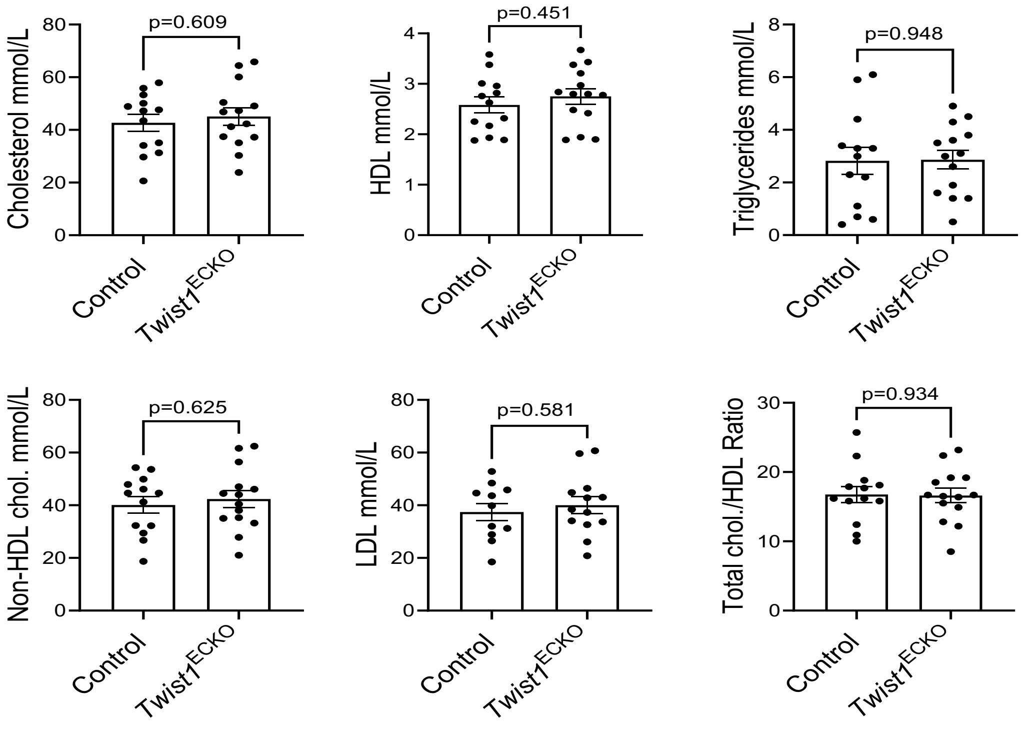

Supplementary Figure 22. *Twist1* does not regulate levels of plasma lipoproteins. *Twist1*^{ECKO} and control mice aged 8 weeks were fed a Western diet for 8 weeks to induce atherosclerotic lesions. Tamoxifen was administered for 5 consecutive days and a Western diet was provided for an additional 6 weeks. Total plasma cholesterol, HDL cholesterol, triglycerides, non-HDL cholesterol, LDL cholesterol and total cholesterol/HDL ratio were measured in *Twist1^{ECKO}* (n=13) and control (n=11) mice. Mean levels +/- standard errors are shown. Differences between means were analysed using an unpaired t-test.

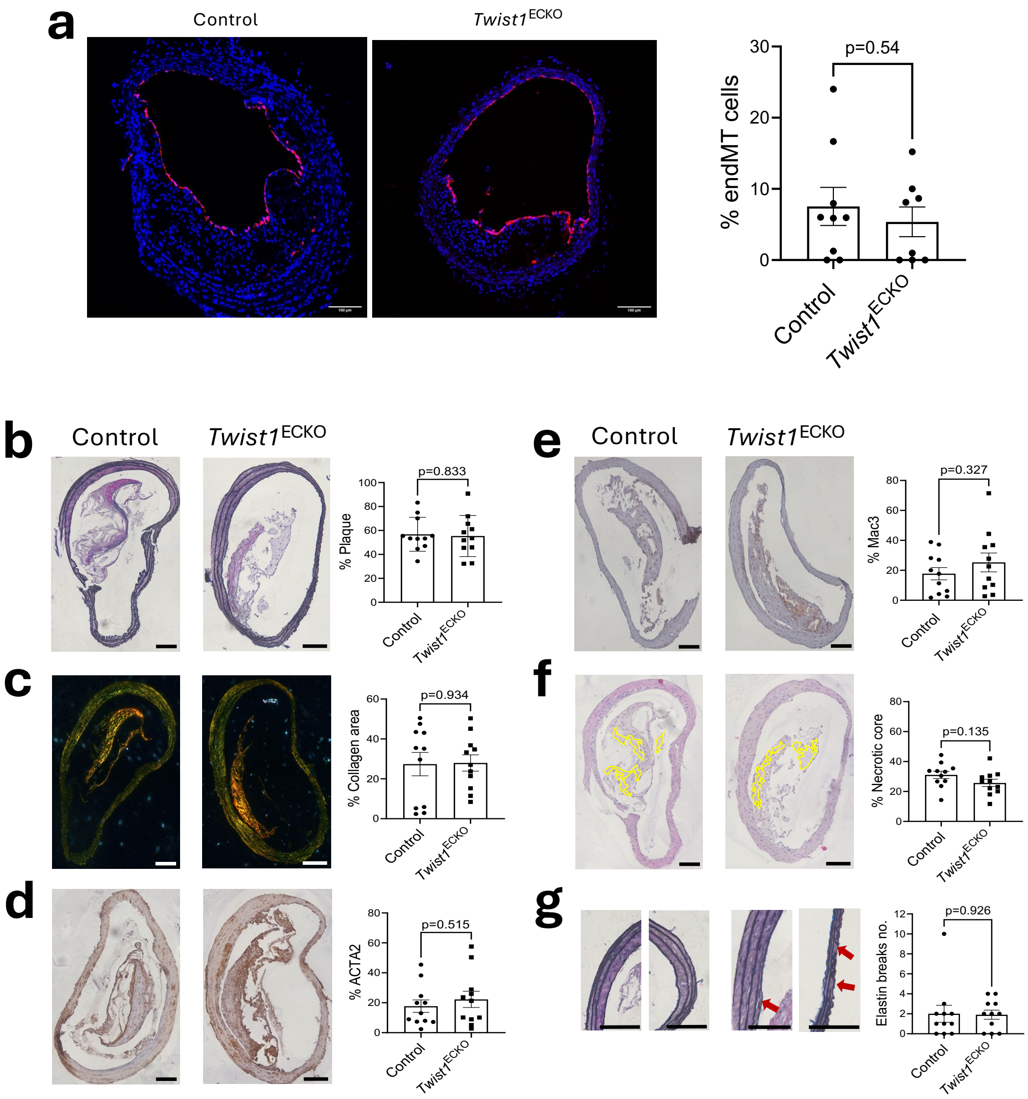

Supplementary Figure 23. *Twist1* does not control EndMT, plaque growth or plaque composition in female mice. (A) Female *Twist1*^{ECKO} (*Twist1*$^{fl/fl}$ *Cdh5*$^{CreERT2/+}$ *ApoE*$^{-/-}$ *Rosa26*$^{TdTomato/TdTomato}$) and control mice (*Twist1*$^{fl/fl}$ *Cdh5*$^{+/+}$ *ApoE*$^{-/-}$ *Rosa26*$^{TdTomato/TdTomato}$) aged 8 weeks were fed a Western diet for 8 weeks to induce atherosclerotic lesions. Tamoxifen was then administered for 5 consecutive days to induce *Twist1* deletion and TdTomato expression in ECs and a Western diet was provided for an additional 6 weeks (totalling 14 weeks of Western diet). The percentage of Rosa26TdTomato^{+} cells that have undergone EndMT was quantified in frozen brachiocephalic sections from *Twist1*^{ECKO} (n=8) and control (n=9) mice. Rosa26TdTomato^{+} cells are shown in red and nuclei are counterstained with DAPI (blue). Representative images are shown (Scale bar=100 µm). (B-F) Female *Twist1*^{ECKO} (*Twist1*$^{fl/fl}$ *Cdh5*$^{CreERT2/+}$ *ApoE*$^{-/-}$) and control mice (*Twist1*$^{fl/fl}$ *Cdh5*$^{+/+}$ *ApoE*$^{-/-}$) aged 8 weeks were fed a Western diet for 8 weeks to induce atherosclerotic lesions. Tamoxifen was then administered for 5 consecutive days to induce *Twist1* deletion in ECs and a Western diet was provided for an additional 6 weeks (totalling 14 weeks of Western diet). Paraffin-embedded sections of brachiocephalic arteries were stained with (B) Miller's elastin stain to quantify plaque size, (C) Picrosirius Red (visualised under polarised light) to quantify collagen content, (D) antibodies against ACTA2 to quantify vSMCs content, (E) antibodies against MAC3 to quantify macrophage content and (F) Hematoxylin and Eosin (H&E) to quantify necrotic core content (highlighted in yellow) in *Twist1*^{ECKO} (n=11) and control (n=11) mice. (G) Magnified view of regions shown in (B) and quantification of elastin breaks number in brachiocephalic arteries from *Twist1*^{ECKO} (n=11) and control (n=11) mice. The red arrows indicate elastin breaks. Representative images are shown (Scale bar=100 µm). Mean values are shown +/- standard errors. Differences between means were analysed using an unpaired t-test.

Supplementary Table 1 PCR Primers for Genotyping

Primer name	Primer sequence
Twist1 Forward	CTTCTCCGTCTGGAGGATGG
Twist1 Reverse	GATGGCGTTTTGGGCACAAGG
Cdh5-Cre Forward	TCGATGCAACGAGTGATGAG
Cdh5-Cre Reverse	AGTGCGTTCGAACGCTAGAG
ApoE Null Forward	GCCTAGCCGAGGGAGAGCCG
ApoE WT Forward	TGTGACTTGGGAGCTCTGCAGC
ApoE Reverse	GCCGCCCCGACTGCATCT
Rosa26TdTomato WT Forward	AAGGGAGCTGCAGTGGAGT
Rosa26TdTomato WT Reverse	CCGAAAATCTGTGGGAAGTC
Rosa26TdTomato Mutant Forward	CTGTTCCTGTACGGCATGG
Rosa26TdTomato Mutant Reverse	GGCATTAAAGCAGCGTATCC

Supplementary Table 2 PCR Primers for qRT-PCR

Organism	Gene	Forward primer	Reverse primer	Purpose
Mus musculus	*Acta2*	CATCATGCGTCTGGACTTGG	AATCTCACGCTCGGCAGTAG	qRT-PCR
Mus musculus	*Cdh5*	TCTTGCCAGCAAACTCTCCT	TTGGAATCAAATGCACATCG	qRT-PCR
Mus musculus	*Cd31*	CGGTGTTCAGCGAGATCC	ACTCGACAGGATGGAAATCAC	qRT-PCR
Mus musculus	*Hprt*	AGTCCCAGCGTCGTGATTAG	TCTCGAGCAAGTCTTTCAGTCC	qRT-PCR
Mus musculus	*Twist1*	ACCTAGATGTCATTGTTTCCAGA	CCACGCCCTGATTCTTGTG	qRT-PCR
Homo sapiens	*AEBP1*	TGAGCGCCAGACAGACGAA	CCTTTCGGGGCTCTTTGTG	qRT-PCR
Homo sapiens	*COL4A1*	TGCGGCTCAAAGGTGACAA	AATCCTACAGAACCCGGCGA	qRT-PCR
Homo sapiens	*DLL4*	TCCAACTGCCCTTCAATTTC	ACTGCAGATGACCCGGTAAG	qRT-PCR
Homo sapiens	*FKBP10*	CATGGGCATGTGTGTCAACG	GAATGAGCCCCGCCAGG	qRT-PCR
Homo sapiens	*HPRT*	TTGGTCAGGCAGTATAATCC	GGGCATATCCTACAACAAAC	qRT-PCR
Homo sapiens	*KDELR3*	TCTGTACCGGGCACTCTACC	ACTTCTTTCCCTTAAGGACTTTGGT	qRT-PCR
Homo sapiens	*MTX2*	AGGGGAGATCACTCATGCTAGG	TGACAGCACTGGTCTACATCC	qRT-PCR
Homo sapiens	*PELP1*	GAGCCCCACAGAGCTATTCC	GGGTCCTGGGTCTGTAACAC	qRT-PCR
Homo sapiens	*RPS7*	TCGTCTTTATCGCTCAGAGGAG	GCACAGCTGTCAGAGTACGG	qRT-PCR

Homo sapiens	*SEC23B*	TACGTGATACAGCGAGGTGC	GCAGGGACTCTTTGAGTGCT	qRT-PCR
Homo sapiens	*TWIST1*	CGGAGACCTAGATGTCATTGTTT	CCACGCCCTGTTTCTTTGAAT	qRT-PCR
Homo sapiens	*USP14*	GGCTTCAGCGCAGTATATTA	CAGATGAGGAGTCTGTCTCT	qRT-PCR
Homo sapiens	*Acta2*	TTTCAGCTTCCCTGAACACCA	GGGCAACACGAAGCTCATTG	qRT-PCR
Homo sapiens	*SNAI1*	TGCAGGACTCTAATCCAGAGTTT	GACAGAGTCCCAGATGAGCA	qRT-PCR
Homo sapiens	*NCAD*	GCACAGATGTGGACAGGATT	CAGCACAAGGATAAGCAGGA	qRT-PCR
Homo sapiens	*AEBP1*	GCTTACTAATGCGCACGCGA	CGTACAACCACAGCACCAAC	ChIP
Homo sapiens	*COL4A1*	CAGTGGAACAGAGCTTCGTAAAAC	TATTGAACCAGTGCTGGAAGGAA	ChIP
Homo sapiens	*COL4A1 (2)*	GCATTGCAAACGCCAGACA	CGTTGGCTGGCTAAATGGGT	ChIP
Homo sapiens	*FKBP10*	CAACTCCAGGCACCATGTTC	CCTGCACCACCAGTAGCAG	ChIP
Homo sapiens	*PELP1*	ATCTGAAGTGCTGGCAACCG	GGTCATCTGGAGAACTTCCCTC	ChIP
Homo sapiens	*SNAI2*	CCGCTTCCCCCTTCCTTTTT	AGCCTCTGGTGTTAATGAGAGC	ChIP
Homo sapiens	Neg	AGTGCCTGCACCCAAGATTT	TGCAAACCTGCTTAACTCCAAC	ChIP

Supplementary Table 3 Antibodies

Antibody	Origin	Dilution	Application	Source	Catalog no.
TWIST1	Mouse	1/200	WB	Santacruz	sc-81417
TWIST1	Mouse	1/200	IF	Abcam	ab175430
Calnexin	Mouse	1/3000	WB	Bd Transduction Laboratories	4178754
PDHX	mouse	1/3000	WB	Santacruz	sc-393644
COL4A1	Rabbit	1/1000 (WB/IF), 1/100 (IHC-f)	WB/IF/IHC-f	Genetex	GTX130215
AEBP1	Rabbit	1/1000 (WB/IF), 1/50 (IHC-f)	WB/IF/IHC-f	Invitrogen	PA5109366
FKBP65	Rabbit	1/1000	WB/IF	Proteintech	12172-1-AP
PELP1	Mouse	1/2000	WB	Proteintech	67050-1
Ki67	Rabbit	1/200	IF	Abcam	AB15580
VE-Cadherin	Mouse	1/300	IF	BD Biosciences	555661

VE-Cadherin	Rabbit	1/250	IF	Abcam	ab33168
SNAI1	Mouse	1/100	IF	Santacruz	sc-271977
CD31	Rabbit	1/500	IF	Abcam	ab182981
anti-Mouse	Goat	1/500	IF	Thermofisher	A-11001
anti-Rabbit	Goat	1/500	IF	Thermofisher	A-11011
anti-Mouse (HRP)	Goat	1/3000	WB	Agilent/Dako	P0447
anti-Rabbit (HRP)	Goat	1/3000	WB	Agilent/Dako	P0448
AF488-CD31	Rat	1/50	FACS	Biolengend	102514
APC-CD45	Rat	1/100	FACS	Biolengend	103112
TruStain FcX™ CD16/32	Rat	1/50	FACS	Biolengend	101320
AF488-ACTA2	Mouse	1/200	IHC-f	Abcam	Ab184675
PELP1	Rabbit	1/400	IHC-f	Invitrogen	PA5-76700
VWF	Rabbit	1/300	IHC-f	DAKO	A0082
ACTA2	Mouse	1/150	IHC-p	DAKO	M0851
ACTA2	Mouse	1/1000	IF	DacoCytomation	M0851
MAC3	Rat	1/75	IHC-p	BD-Pharmigen	550292
Anti-FLAG	Rabbit	10ug	ChIP	Cell signalling	14793S
IgG	Rabbit	10ug	ChIP	Diagenode	C15410206
SM22A	Rabbit	1/100	IF	Abcam	Ab14106
NCAD	Rabbit	1/100	IF	Cell signaling	13116

SUPPLEMENTARY REFERENCES

1. Kan, H., *et al.* Single-cell transcriptome analysis reveals cellular heterogeneity in the ascending aortas of normal and high-fat diet-fed mice. *Experimental & Molecular Medicine* **53**, 1379-1389 (2021).
2. Bankhead, P., *et al.* QuPath: Open source software for digital pathology image analysis. *Scientific Reports* **7**, 16878 (2017).
3. Schmidt, U., Weigert, M., Broaddus, C. & Myers, E.W. Cell Detection with Star-convex Polygons. in *International Conference on Medical Image Computing and Computer-Assisted Intervention* (2018).

UNPROCESSED WESTERN BLOT IMAGES

Supplementary Fig. 19A

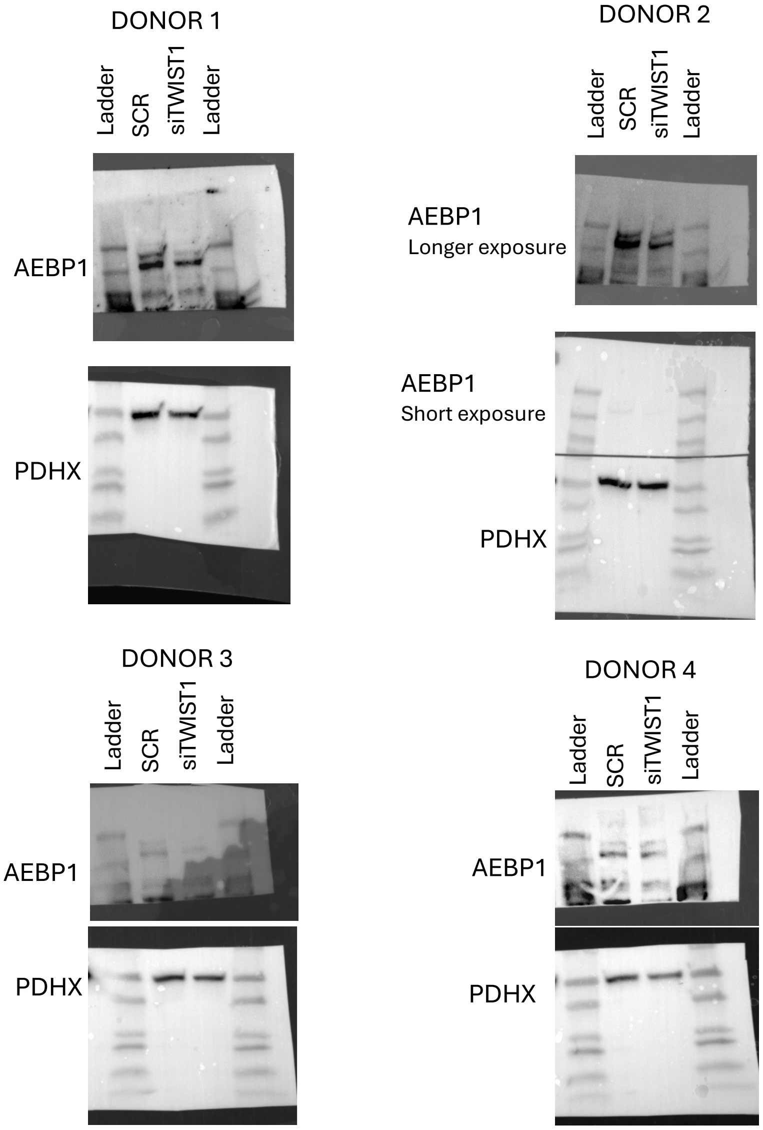

Supplement: Supplementary file 1 — Supplementary Information [file 41467_2026_69808_MOESM1_ESM.pdf]
