# Supplementary material for: TWIST1 drives endothelial-to-mesenchymal-transition to stabilize atherosclerotic plaques
Source: Nat Commun. 2026 Feb 18;17:2905. doi: 10.1038/s41467-026-69808-z (PMC13031644; doi:10.1038/s41467-026-69808-z)
Supplement: Supplementary file 2 — Reporting Summary [file 41467_2026_69808_MOESM2_ESM.pdf]

Corresponding author(s): Paul Evans
Last updated by author(s): Jan 12, 2026

Reporting Summary

Nature Portfolio wishes to improve the reproducibility of the work that we publish. This form provides structure for consistency and transparency in reporting. For further information on Nature Portfolio policies, see our Editorial Policies and the Editorial Policy Checklist.

Statistics

For all statistical analyses, confirm that the following items are present in the figure legend, table legend, main text, or Methods section.

n/a	Confirmed	
☐	☒	The exact sample size (*n*) for each experimental group/condition, given as a discrete number and unit of measurement
☐	☒	A statement on whether measurements were taken from distinct samples or whether the same sample was measured repeatedly
☐	☒	The statistical test(s) used AND whether they are one- or two-sided<br>*Only common tests should be described solely by name; describe more complex techniques in the Methods section.*
☐	☒	A description of all covariates tested
☐	☒	A description of any assumptions or corrections, such as tests of normality and adjustment for multiple comparisons
☐	☒	A full description of the statistical parameters including central tendency (e.g. means) or other basic estimates (e.g. regression coefficient) AND variation (e.g. standard deviation) or associated estimates of uncertainty (e.g. confidence intervals)
☐	☒	For null hypothesis testing, the test statistic (e.g. *F*, *t*, *r*) with confidence intervals, effect sizes, degrees of freedom and *P* value noted<br>*Give P values as exact values whenever suitable.*
☐	☒	For Bayesian analysis, information on the choice of priors and Markov chain Monte Carlo settings
☐	☒	For hierarchical and complex designs, identification of the appropriate level for tests and full reporting of outcomes
☒	☐	Estimates of effect sizes (e.g. Cohen's *d*, Pearson's *r*), indicating how they were calculated

Our web collection on statistics for biologists contains articles on many of the points above.

Software and code

Policy information about availability of computer code

Data collection	No software was used.
Data analysis	Statistical analysis was carried out using Prism v10

For manuscripts utilizing custom algorithms or software that are central to the research but not yet described in published literature, software must be made available to editors and reviewers. We strongly encourage code deposition in a community repository (e.g. GitHub). See the Nature Portfolio guidelines for submitting code & software for further information.

Data

Policy information about availability of data

All manuscripts must include a data availability statement. This statement should provide the following information, where applicable:

- Accession codes, unique identifiers, or web links for publicly available datasets
- A description of any restrictions on data availability
- For clinical datasets or third party data, please ensure that the statement adheres to our policy

The fully annotated scRNA-seq and bulk RNA-seq generated in this study have been deposited in the Gene Expression Omnibus database under accession code GSE293412 [https://www.ncbi.nlm.nih.gov/geo/query/acc.cgi?acc=GSE293412]. The quantitative data generated from analysis of mice and human cells are provided in the Source Data file. The human data is protected due to privacy laws and would be shared in group form upon request from a qualified academic investigator for the sole purpose of replicating the procedures and results presented in the article and providing that the data transfer is in agreement with European Union legislation on the general data protection regulation and decisions by the ethical review board of Sweden, the Region Skåne and the Lund

University. Professor Isabel Goncalves (Isabel.Goncalves@med.lu.se) may be contacted for access to human data. Data regarding living subjects cannot be publicly available due to the sensitive nature of the data regulated by GDPR.

Research involving human participants, their data, or biological material

Policy information about studies with human participants or human data. See also policy information about sex, gender (identity/presentation), and sexual orientation and race, ethnicity and racism.

Reporting on sex and gender	All participants were included if they were eligible for carotid endarterectomy due to a stenosis degree >80% without previous cerebrovascular symptoms or to a stenosis degree >70% with ischemic stroke, transient ischemic attack, or amaurosis fugax.
Reporting on race, ethnicity, or other socially relevant groupings	We did not perform subanalyses based on race, ethnicity or other socially-relevant groupings.
Population characteristics	The clinical characteristics of the patients are detailed in JACC 2023; 81: 2213-2227.
Recruitment	Carotid endarterectomy samples were collected. The indications for surgery were: 1) asymptomatic carotid stenosis with a stenosis degree >80% or 2) cerebrovascular symptoms (ischemic stroke, transient ischemic attack, or amaurosis fugax within one month prior to surgery) and a carotid plaque with stenosis degree >70%. For follow-up analysis, information regarding postoperative cardiovascular events (myocardial infarction, unstable angina, stroke (ipsilateral and contralateral events), transient ischemic attack, amaurosis fugax, vascular interventions (including carotid endarterectomy/stenting, coronary artery bypass grafting/percutaneous coronary artery intervention) and CV death was acquired from the Swedish Cause of Death and National inpatient Health Registers. The participants were followed until events or end of follow-up by the 31st December 2015.
Ethics oversight	Human plaque bulk RNA-seq and immunostaining of TWIST1 were performed on carotid plaques obtained from the CPIP biobank (Region Skåne, Malmö, Sweden). All patients have given written informed consent, and the study follows the declaration of Helsinki. The study protocol has been approved by the local ethical committee in Lund and the Swedish ethical committee (472/2005, 2014/904, 60/2008, 2012/209).

Note that full information on the approval of the study protocol must also be provided in the manuscript.

Field-specific reporting

Please select the one below that is the best fit for your research. If you are not sure, read the appropriate sections before making your selection.

☒ Life sciences ☐ Behavioural & social sciences ☐ Ecological, evolutionary & environmental sciences

For a reference copy of the document with all sections, see nature.com/documents/nr-reporting-summary-flat.pdf

Life sciences study design

All studies must disclose on these points even when the disclosure is negative.

Sample size	Sample size for experiments on mice were determined by Power Calculations based on the effects of TWIST1 observed in preminiary experiments.
Data exclusions	No data were excluded from the analyses.
Replication	All experiments were replicated on multiple occasions and this is detailed in the figure legends.
Randomization	Experimental mice were bred from multiple sets of heterozygous parents. Twist1ECKO mice and control littermates were obtained from the same litters. Allocation to experimental groups was determined by the random inheritance of the Cre allele, which occurs at approximately 50% frequency. Mice carrying the Cre allele were designated as Twist1ECKO, whereas mice lacking the Cre allele served as controls. Genotyping was performed to identify the presence or absence of the Cre allele, and mice were assigned to experimental groups accordingly.
Blinding	The Investigators were blinded to the identity of samples from Twist1ECKO and control mice during experiments and outcome assessment. Plaque analysis was carried out by Dr Ayllon and then confirmed through requantification by Dr Tian. Both analyses gave closely similar results.

Reporting for specific materials, systems and methods

We require information from authors about some types of materials, experimental systems and methods used in many studies. Here, indicate whether each material, system or method listed is relevant to your study. If you are not sure if a list item applies to your research, read the appropriate section before selecting a response.

Materials & experimental systems

n/a	Involved in the study
☐	☒ Antibodies
☐	☒ Eukaryotic cell lines
☒	☐ Palaeontology and archaeology
☐	☒ Animals and other organisms
☒	☐ Clinical data
☒	☐ Dual use research of concern
☒	☐ Plants

Methods

n/a	Involved in the study
☒	☐ ChIP-seq
☒	☐ Flow cytometry
☒	☐ MRI-based neuroimaging

Antibodies

Antibodies used

Antibody Origin Dilution Application Source Catalog no.
1. TWIST1 Mouse 1/200 WB Santacruz sc-81417
2. TWIST1 Mouse 1/200 IF Abcam ab175430
3. Calnexin Mouse 1/3000 WB Bd Transduction
Laboratories 4178754
4. PDHX mouse 1/3000 WB Santacruz sc-393644
5. COL4A1 Rabbit 1/1000 (WB/IF), 1/100 (IHC-f) WB/IF/IHC-f Genetex GTX130215
6. AEBP1 Rabbit 1/1000 (WB/IF), 1/50 (IHC-f) WB/IF/IHC-f Invitrogen PA5109366
7. FKBP65 Rabbit 1/1000 WB/IF Proteintech 12172-1-AP
8. PELP1 Mouse 1/2000 WB Proteintech 67050-1
9. Ki67 Rabbit 1/200 IF Abcam AB15580
10. VE-Cadherin Mouse 1/300 IF BD Biosciences 555661
11. VE-Cadherin Rabbit 1/250 IF Abcam ab33168
12. SNAI1 Mouse 1/100 IF Santacruz sc-271977
13 .CD31 Rabbit 1/500 IF Abcam ab182981
14 anti-Mouse Goat 1/500 IF Thermofisher A-11001; anti-Rabbit Goat 1/500 IF Thermofisher A-11011
15. anti-Mouse (HRP) Goat 1/3000 WB Agilent/Dako P0447
16. anti-Rabbit (HRP) Goat 1/3000 WB Agilent/Dako P0448
17. AF488-CD31 Rat 1/50 FACS Biolengend 102514
18. APC-CD45 Rat 1/100 FACS Biolengend 103112
19. TruStain FcX™ CD16/32
20. Rat 1/50 FACS Biolengend 101320
21. AF488-ACTA2 Mouse 1/200 IHC-f Abcam Ab184675
22. ELP1 Rabbit 1/400 IHC-f Invitrogen PA5-76700
23. VWF Rabbit 1/300 IHC-f DAKO A0082
24. ACTA2 Mouse 1/150 IHC-p DAKO M0851
25. ACTA2 Mouse 1/1000 IF DacoCytomation M0851
26. MAC3 Rat 1/75 IHC-p BD-Pharmigen 550292
27. Anti-FLAG Rabbit 10ug ChIP Cell signalling 14793S
28. IgG Rabbit 10ug ChIP Diagenode C15410206
29. SM22A Rabbit 1/100 IF Abcam Ab14106
30. NCAD Rabbit 1/100 IF Cell signaling 13116

Validation

Primary antibodies were validated by gene silencing in cultured cells - this is described in the manuscript.

Eukaryotic cell lines

Policy information about cell lines and Sex and Gender in Research

Cell line source(s)

HEK293T cells were obtained from ATCC

Authentication

Authentication was not carried out.

Mycoplasma contamination

Testing for mycoplasma was carried out routinely.

Commonly misidentified lines
(See ICLAC register)

None.

Animals and other research organisms

Policy information about studies involving animals; ARRIVE guidelines recommended for reporting animal research, and Sex and Gender in Research

Laboratory animals	All mice were on a C57BL/6J background. Twist1fl/fl mice were obtained from Dr Akiko Mammoto (Medical College of Wisconsin, USA). Rosa26TdTomato were kindly supplied by Professor Nicola Smart (University of Oxford, UK). Cdh5CreERT2/+ and ApoE-/- colonies were obtained from Professor Sheila Francis (University of Sheffield, UK). Mice aged 8 weeks old were given a Western diet (TD.88137, Envigo) for 14 weeks. Male and female mice were studied.
Wild animals	The study did not involve wild animals
Reporting on sex	Male and female mice were studied.
Field-collected samples	Samples were not collected from the field.
Ethics oversight	Animal care and experimental procedures were carried out under licenses issued by the UK Home Office, and approval from the ethical committee of the University of Sheffield, UK was obtained.

Note that full information on the approval of the study protocol must also be provided in the manuscript.

Plants

Seed stocks	NA
Novel plant genotypes	NA
Authentication	NA
