# Supplementary figures and images for: TWIST1 drives endothelial-to-mesenchymal-transition to stabilize atherosclerotic plaques
Source: Nat Commun. 2026 Feb 18;17:2905. doi: 10.1038/s41467-026-69808-z (PMC13031644; doi:10.1038/s41467-026-69808-z)

**UNPROCESSED WESTERN BLOT IMAGES**

**Fig 3A**

**Fig 4F**

**Fig 5E**

**
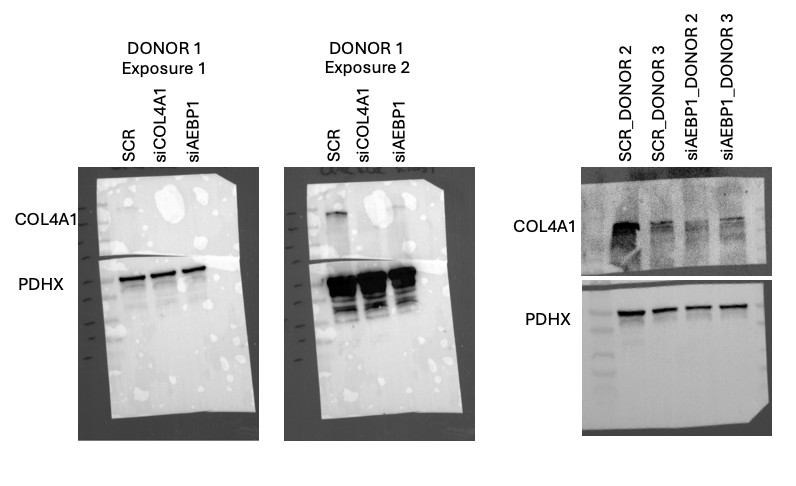
**

Supplement: Supplementary file 5 — Source Data-Uncropped Western Blots [file 41467_2026_69808_MOESM5_ESM.docx]
